# Supplementary material for: Enhancing museum visitor forecasting using deep learning and sentiment analysis: A transformer-based approach for sustainable management
Source: PLoS One. 2025 Nov 11;20(11):e0335623. doi: 10.1371/journal.pone.0335623 (PMC12604778; doi:10.1371/journal.pone.0335623)
Supplement: Attachment — Submitted filename: Response to Reviewers_plosone.docx [file pone.0335623.s002.docx]

**Response to Reviewers**

**Enhancing Museum Visitor Forecasting Using Deep Learning and Sentiment Analysis: A Transformer-Based Approach for Sustainable Management**

**Reviewer #1:**

1. How was the model validated to ensure it generalizes well across different museum environments or geographic locations? Could the authors provide further details on the external validation process, if any, such as using data from museums not included in the training dataset?

**[Response]**

We appreciate the reviewer’s insightful comment regarding model generalizability across varying museum contexts. In response, we clarify that in addition to the 5-fold cross-validation implemented for internal validation, we conducted external validation using a holdout sample comprising museums from a distinct administrative region not included in the training set. Specifically, the model was trained on museums located in the Seoul Capital Area and validated on museums located in provincial regions such as Chungcheong, Gyeongsang, and Jeolla provinces. This approach enabled us to test the model’s robustness and predictive capacity beyond the urban-centric training dataset.

The performance of the upgrade predictive model was assessed using metrics such as MAE (Mean Absolute Error), RMSE (Root Mean Square Error), and R² (Coefficient of Determination), both for internal cross-validation and the external holdout dataset. The results demonstrated consistent predictive accuracy across diverse geographic museum settings, thereby supporting the model’s applicability in a variety of museum environments. Details of this external validation process have now been included in Section 3.4 of the revised manuscript.

**[Revision]**

To ensure the robustness and generalizability of the proposed model across varying museum environments and geographic locations, we implemented both internal cross-validation and external holdout validation procedures. For internal validation, we employed a K-fold cross-validation technique (K=5), where the dataset was partitioned into five equal subsets. Each subset alternated as the validation set while the remaining four were used for training. This method ensures that all data points are utilized for both training and testing, thereby minimizing overfitting and improving the generalizability of the model across diverse patterns within the dataset [21,22].

In addition to this internal validation, external validation was conducted to test the model’s performance on geographically distinct museums. The training dataset consisted of museums located in the Seoul Capital Region (Seoul, Incheon, Gyeonggi), while the validation set included museums from non-metropolitan areas, such as Chungcheong, Gyeongsang, and Jeolla provinces. This geographic separation allowed us to test whether the deep learning model could accurately predict visitor numbers for museums operating under different regional contexts, infrastructure conditions, and population densities.

Performance metrics including MAE (Mean Absolute Error), RMSE (Root Mean Square Error), and R² (Coefficient of Determination) were calculated for both internal and external evaluations. The upgrade predictive model demonstrated robust accuracy not only within the cross-validation folds but also when applied to unseen, regionally distinct museum data. This indicates that the model is effective in capturing underlying patterns that are not limited to a specific locality, thereby enhancing its utility for national-level museum management and policy planning. By integrating structured museum attributes, operational metrics, and public sentiment data, the model provides a flexible and scalable framework that can be applied across diverse institutional settings to support sustainable museum management.

2. It would be beneficial to see a comparative analysis with traditional forecasting models. How do the proposed deep learning models perform in comparison to these more traditional approaches in terms of accuracy and computational efficiency?

**[Response]**

We sincerely thank the reviewer for raising this important point. To address the need for comparative analysis with traditional forecasting models, we have included Linear Regression (LR) and Random Forest Regression (RFR) as baseline models in our empirical evaluation. These models were selected for their widespread use in time-series forecasting and interpretability.

Both LR and RFR were applied to the same dataset and evaluated using the same metrics as the proposed deep learning models (RNN, LSTM, CNN, GAN, Transformer). The performance comparison—presented in Tables 4 through 8—clearly demonstrates that while traditional models offer faster computation and ease of interpretation, they are less capable of capturing complex nonlinear patterns and public sentiment dynamics present in both structured and unstructured data.

In particular, deep learning models such as Transformer and LSTM significantly outperformed traditional models across all evaluation metrics (R², MSE, RMSE, MSLE, MAPE). For example, in the full model incorporating sentiment data (NEWS and COMMENTS), the Transformer achieved an R² of 0.7579 compared to 0.5834 for LR and 0.6227 for RFR. Similar trends were observed for all error-based metrics.

This finding supports the view that deep learning models are better suited for high-dimensional, context-aware forecasting tasks, such as predicting museum visitor numbers influenced by external media sentiment. We have revised Sections 3.5 and 3.6 accordingly and added the performance results of the baseline models in Tables 4 through 8 to reflect this comparative analysis.

**[Revision]**

**4. Analysis Results**

Table 3 presents the R-squared results for predicting museum visitor numbers using various deep learning algorithms, including RNN, GAN, CNN, LSTM, and Transformer models. The analysis compares the performance of the base model, which relies solely on structured museum-related data, with models that incorporate additional variables derived from sentiment analysis of museum-related news articles (NEWS) and user comments (COMMENTS). Furthermore, the performance of models that integrate both NEWS and COMMENTS is evaluated to assess the impact of combining multiple external opinion factors. The R-squared values, which measure the proportion of variance in the dependent variable (museum visitor numbers) explained by the independent variables, provide critical insights into the predictive performance of each model. A higher R-squared value indicates stronger explanatory power and better predictive accuracy.

In addition to deep learning algorithms, traditional forecasting models such as Linear Regression (LR) and Random Forest (RF) were also evaluated to provide a comparative baseline. The base model, which does not include sentiment analysis data, exhibits relatively moderate predictive power across all algorithms, with R-squared values ranging from 0.5213 for Linear Regression and 0.5638 for Random Forest to 0.6250 for the RNN model and 0.7041 for the Transformer model. These results suggest that while traditional models provide reasonable explanatory power based on structured variables alone, they are generally outperformed by deep learning models capable of capturing complex, non-linear dependencies in the data. Among the deep learning models, the Transformer model achieves the highest R-squared in the base model configuration. This result suggests that the Transformer's ability to capture complex, non-linear relationships in the data contributes to its superior baseline performance, which aligns with previous research on its effectiveness in time-series forecasting.

When sentiment analysis data from museum-related news articles (NEWS) is added, there is a noticeable improvement in predictive performance across all models. The R-squared values increase, with the Transformer model showing an improvement from 0.7041 to 0.7245, and the LSTM model rising from 0.6874 to 0.7038. Likewise, the traditional models also demonstrate gains: the R-squared of Linear Regression improves from 0.5213 to 0.5425, and that of Random Forest increases from 0.5638 to 0.5880. This enhancement indicates that public sentiment, as reflected in news articles, significantly influences visitor behavior. Positive media coverage likely stimulates interest, while negative sentiment may deter potential visitors, suggesting that public perception plays a crucial role in shaping visitor patterns.

The inclusion of sentiment analysis from user comments (COMMENTS) also results in performance gains, although the improvements are slightly less pronounced compared to the NEWS-enhanced models. For instance, the RNN model improves from 0.6250 to 0.6681, and the Transformer model increases from 0.7041 to 0.7387. Linear Regression and Random Forest also show consistent improvements with comment-based sentiment data, reaching R-squared values of 0.5587 and 0.6032, respectively. This finding suggests that while user-generated comments do influence visitor behavior, their impact may be less immediate or direct compared to news articles. This could be attributed to the fragmented and subjective nature of comments, which may vary widely in tone and content, making it more challenging to capture consistent trends.

The most significant improvements are observed when both NEWS and COMMENTS are incorporated into the models. The Transformer model achieves the highest R-squared value of 0.7579, followed closely by LSTM at 0.7496 and CNN at 0.7238. Traditional models also benefit from the dual integration of sentiment variables: Linear Regression rises to 0.5834, and Random Forest reaches 0.6227. This result highlights the complementary effect of combining different sources of external opinion data. The synergy between formal news content and informal user feedback enhances the models' ability to capture the multifaceted nature of public interest in museums. The integration of these diverse data sources allows the models to account for both structured museum-related factors and dynamic, real-time external influences, leading to more accurate visitor number predictions.

The results of this study provide several key insights and implications. First, the superior performance of the Transformer and LSTM models is evident across all configurations. The Transformer consistently outperforms other models, reflecting its strong capability to process complex, multivariate time-series data and capture long-range dependencies. LSTM, known for its effectiveness in handling sequential data, also demonstrates robust performance, particularly when sentiment data is integrated. These findings suggest that both models are well-suited for tasks involving the prediction of museum visitor numbers, especially in contexts where both historical data and real-time external factors are relevant.

Second, while traditional models such as LR and RF show lower overall predictive power, the integration of sentiment data significantly enhances their performance as well. This demonstrates that even simpler models can benefit from incorporating external opinion data, particularly in resource-constrained settings where deep learning models may not be feasible.

Third, the importance of external opinion data is clearly underscored. The results highlight the value of incorporating unstructured data sources such as news articles and user comments. Models that leverage opinion mining exhibit significant improvements in predictive accuracy, suggesting that visitor behavior is influenced not only by internal museum factors, such as exhibition content and operational schedules, but also by external social dynamics, including media coverage and public discourse. This insight emphasizes the need for museums to monitor and respond to public sentiment as part of their strategic management efforts.

From a practical perspective, these findings have important implications for museum management. Understanding the impact of public sentiment can help museums tailor their marketing strategies, improve public engagement, and anticipate fluctuations in visitor numbers more effectively. Additionally, the ability to predict visitor trends with greater accuracy supports resource optimization, event planning, and sustainable operational management. Museums can use these predictive insights to allocate resources more efficiently, schedule staff appropriately, and design targeted promotional campaigns that resonate with current public interests.

In conclusion, the integration of sentiment analysis data into deep learning models significantly enhances the prediction of museum visitor numbers. The comparative evaluation further reveals that while advanced models like Transformer and LSTM offer the highest performance, traditional models also see measurable gains from opinion mining. The superior performance of the Transformer and LSTM models, combined with the added value of external opinion data, offers a comprehensive approach to understanding and forecasting visitor behavior. This approach contributes to more effective and sustainable museum management, enabling institutions to adapt to changing visitor dynamics and optimize their operations in response to both historical trends and real-time public sentiment.

**Table 3. Prediction of Museum Visitor Numbers R-squared results**

| Model | Regressor | R-squared | Regressor | R-squared | Regressor | R-squared | Regressor | R-squared | Regressor | R-squared | Regressor | R-squared | Regressor | R-squared |
| --- | --- | --- | --- | --- | --- | --- | --- | --- | --- | --- | --- | --- | --- | --- |
| Base model | LR | 0.5213 | RFL | 0.5638 | RNN | 0.6250 | GAN | 0.6453 | CNN | 0.6688 | LSTM | 0.6874 | Transformer | 0.7041 |
| Added  NEWS |  | 0.5425 |  | 0.5880 |  | 0.6547 |  | 0.6692 |  | 0.6871 |  | 0.7038 |  | 0.7245 |
| Added COMMENTS |  | 0.5587 |  | 0.6032 |  | 0.6681 |  | 0.6875 |  | 0.6954 |  | 0.7192 |  | 0.7387 |
| Added  NEWS & COMMENTS |  | 0.5834 |  | 0.6227 |  | 0.7012 |  | 0.7133 |  | 0.7238 |  | 0.7496 |  | 0.7579 |

Table 4 displays the Mean Squared Error (MSE) outcomes for predicting museum visitor numbers using various deep learning algorithms, including RNN, GAN, CNN, LSTM, and Transformer models. The analysis compares the performance of the base model, which relies exclusively on structured museum related data, with models that incorporate sentiment analysis variables derived from museum-related news articles (NEWS) and user comments (COMMENTS). Additionally, the combined effect of integrating both NEWS and COMMENTS is assessed to understand how external opinion data enhances predictive performance.

MSE is a fundamental metric in evaluating regression models as it quantifies the average squared deviation between predicted and actual values. A lower MSE signifies improved model accuracy, indicating smaller prediction errors. Since MSE penalizes larger errors more heavily, it is particularly effective for measuring the precision of forecasting models, especially in time-series contexts such as museum visitor predictions.

In addition to deep learning algorithms, traditional machine learning models such as Linear Regression (LR) and Random Forest (RF) were also assessed to provide a comparative baseline. The base model, which excludes sentiment analysis data, demonstrates moderate predictive capability across all models, with MSE values ranging from 0.3142 for LR and 0.2981 for RF to 0.2852 for RNN and 0.2084 for the Transformer model. The Transformer model exhibits the most accurate baseline predictions, achieving the lowest MSE among all algorithms. This superior performance can be attributed to the Transformer's sophisticated architecture, particularly its attention mechanism, which enables it to identify complex, non-linear dependencies in the data and effectively model long-term temporal patterns. These capabilities contribute significantly to its higher baseline performance.

When sentiment analysis data from museum-related news articles (NEWS) is introduced, all models show noticeable enhancements in predictive accuracy. For example, the MSE for the Transformer model decreases from 0.2084 to 0.1876, while the LSTM model improves from 0.2241 to 0.2082. Similarly, traditional models also show improvement: the MSE of LR drops from 0.3142 to 0.2938, and RF from 0.2981 to 0.2756. These improvements highlight the substantial role of media sentiment in influencing visitor behavior. Positive news coverage likely attracts more visitors by enhancing public interest and fostering favorable perceptions of museums, while negative reports may discourage attendance. This pattern aligns with prior studies indicating that external socio-cultural factors, such as media narratives, significantly affect tourism demand and public engagement with cultural institutions.

The addition of sentiment analysis from user comments (COMMENTS) also leads to performance improvements, though the gains are slightly less pronounced compared to the NEWS-enhanced models. For instance, the RNN model’s MSE decreases from 0.2852 to 0.2454, and the Transformer’s MSE improves from 0.2084 to 0.1654. Likewise, LR and RF show further reductions to 0.2812 and 0.2603, respectively. This suggests that user-generated comments, despite their informal and subjective nature, offer valuable insights into public sentiment and visitor intentions. However, the impact of such data may be less consistent than news articles due to variability in comment quality, context, and emotional tone.

The most significant enhancements in model performance occur when both NEWS and COMMENTS are integrated. The Transformer model achieves the lowest MSE of 0.1492, followed by the LSTM model at 0.1694 and CNN at 0.1862. The performance of traditional models also improves in this integrated configuration, with LR recording 0.2657 and RF achieving 0.2429. This indicates that combining different sources of external opinion data creates a synergistic effect, allowing the models to capture a broader spectrum of public sentiment. The integration of formal media narratives with informal user feedback enables the models to account for both macro-level trends (reflected in news articles) and micro-level sentiments (expressed in user comments), thus improving predictive accuracy.

Key findings and implications emerge from these results. Firstly, the Transformer and LSTM models consistently deliver superior predictive performance compared to other algorithms, regardless of the model configuration. The Transformer's advanced design, characterized by its ability to process large, complex datasets through parallelization and attention-based mechanisms, makes it exceptionally well-suited for time-series forecasting tasks influenced by dynamic external variables. The LSTM model also demonstrates strong performance, particularly in handling sequential data where long-term dependencies are critical. Its gated structure enables it to retain and process relevant information over extended timeframes, enhancing its ability to forecast visitor trends influenced by both historical patterns and current events.

Secondly, although traditional models such as LR and RF underperform relative to deep learning models, the results clearly indicate that these models also benefit significantly from the integration of sentiment data. The consistent decline in their MSE values across NEWS, COMMENTS, and combined configurations demonstrates that even relatively simple algorithms can exploit external opinion variables for better forecasting. This is particularly relevant in scenarios where computational constraints limit the use of complex deep learning models.

Thirdly, these results highlight the importance of integrating external opinion data into predictive models. The incorporation of sentiment analysis from both news articles and user comments significantly reduces prediction errors, as evidenced by substantial decreases in MSE across all models. This suggests that visitor behavior is shaped not only by internal factors, such as museum exhibitions, programs, and operational schedules, but also by external social dynamics, including public discourse and media representation. By capturing and quantifying these external influences, the models become more robust and reliable, offering more precise forecasts that can support strategic decision making within museum management.

From a practical standpoint, these findings offer valuable insights for sustainable museum management. The improved predictive accuracy achieved through sentiment analysis integration enables museums to anticipate fluctuations in visitor numbers more effectively. This facilitates proactive decision making in resource allocation, staffing, event planning, and marketing strategy. Museums can leverage these insights to optimize their outreach efforts, design more engaging public programs, and tailor promotional activities based on real-time public sentiment. Furthermore, accurate forecasting supports the development of sustainable operational practices, ensuring efficient resource management and enhancing the overall visitor experience.

Namely, the integration of sentiment analysis data from news articles and user comments into deep learning models significantly enhances the ability to predict museum visitor numbers. The comparative results also reveal that traditional models, while less powerful in isolation, can achieve meaningful improvements when supplemented with external sentiment data. The Transformer's and LSTM’s superior performance, combined with the added predictive power of external opinion data, provides a comprehensive framework for understanding and forecasting visitor behavior. This integrated approach not only improves predictive accuracy but also contributes to more informed, data-driven, and sustainable museum management practices, enabling institutions to adapt effectively to evolving visitor dynamics.

**Table 4. Prediction of Museum Visitor Numbers MSE results**

| Model | Regressor | MSE | Regressor | MSE | Regressor | MSE | Regressor | MSE | Regressor | MSE | Regressor | MSE | Regressor | MSE |
| --- | --- | --- | --- | --- | --- | --- | --- | --- | --- | --- | --- | --- | --- | --- |
| Base model | LR | 0.3142 | RFL | 0.2981 | RNN | 0.2852 | GAN | 0.2681 | CNN | 0.2466 | LSTM | 0.2241 | Transformer | 0.2084 |
| Added  NEWS |  | 0.2938 |  | 0.2756 |  | 0.2631 |  | 0.2475 |  | 0.2271 |  | 0.2082 |  | 0.1876 |
| Added COMMENTS |  | 0.2812 |  | 0.2603 |  | 0.2454 |  | 0.2692 |  | 0.2093 |  | 0.1894 |  | 0.1654 |
| Added  NEWS & COMMENTS |  | 0.2657 |  | 0.2429 |  | 0.2267 |  | 0.2431 |  | 0.1862 |  | 0.1694 |  | 0.1492 |

Table 5 presents the Root Mean Squared Error (RMSE) results for predicting museum visitor numbers using various deep learning algorithms, including RNN, GAN, CNN, LSTM, and Transformer models, as well as traditional regression approaches such as Linear Regression (LR) and Random Forest Regressor (RFR). The analysis compares the performance of the base model, which relies solely on structured museum-related data, with models that incorporate additional sentiment analysis variables derived from museum-related news articles (NEWS) and user comments (COMMENTS). Furthermore, the combined effect of integrating both NEWS and COMMENTS is evaluated to determine the extent to which external opinion factors enhance the model's predictive accuracy.

RMSE is a commonly used performance metric in regression analysis that measures the square root of the average squared differences between predicted and actual values. As RMSE retains the same units as the dependent variable (in this case, the log-transformed museum visitor numbers), it provides an intuitive measure of prediction error magnitude. Lower RMSE values indicate better model performance, reflecting more accurate predictions with smaller deviations from actual observations.

In the base model, which does not include sentiment analysis data, the RMSE values range from 0.2664 for the RNN model to 0.1884 for the Transformer model. In comparison, traditional models such as LR and RFR exhibit relatively higher RMSE values of 0.3343 and 0.3253, respectively, indicating their limited predictive capability in capturing non-linear patterns inherent in visitor trends. Among the evaluated algorithms, the Transformer model consistently demonstrates superior baseline performance, achieving the lowest RMSE. This result highlights the Transformer's effectiveness in modeling complex, non-linear relationships within time-series data, attributed to its attention mechanism, which efficiently captures long-range dependencies and temporal patterns.

The incorporation of sentiment analysis data from museum-related news articles (NEWS) leads to notable improvements in predictive performance across all models. For example, the RMSE for the Transformer model decreases from 0.1884 to 0.1618, while the LSTM model shows an improvement from 0.2063 to 0.1878. Traditional models also benefit from the inclusion of NEWS variables, with LR improving from 0.3343 to 0.3165 and RFR from 0.3253 to 0.2993, though the performance gap compared to deep learning models remains substantial. These reductions indicate that public sentiment, as reflected in news coverage, plays a significant role in influencing visitor behavior. Positive media exposure likely enhances public interest and engagement, thereby increasing visitor numbers, while negative news may discourage attendance. This observation aligns with existing research highlighting the impact of media narratives on public behavior and tourism trends.

Similarly, the inclusion of sentiment analysis from user comments (COMMENTS) also results in performance gains, albeit to a slightly lesser extent compared to the NEWS-enhanced models. For instance, the RMSE for the RNN model decreases from 0.2664 to 0.2271, and for the Transformer model, it improves from 0.1884 to 0.1408. Traditional models also reflect meaningful reductions, with LR improving to 0.3020 and RFR to 0.2819. This suggests that while user-generated comments provide valuable insights into public perceptions and experiences, their influence on visitor behavior may be more diffuse and less immediate than that of news articles. The subjective and varied nature of online comments could contribute to this difference, as they reflect diverse individual opinions rather than cohesive media narratives.

The most significant reductions in RMSE are observed when both NEWS and COMMENTS are integrated into the models. The Transformer model achieves the lowest RMSE of 0.1277, followed closely by the LSTM model at 0.1477 and CNN at 0.1664. Even traditional models show notable improvement in this configuration, with LR achieving 0.2842 and RFR 0.2641. These results underscore that the benefit of external sentiment data is not limited to deep learning models, but that more sophisticated architectures are better equipped to extract and utilize this information effectively**.** This finding underscores the complementary effect of combining multiple external opinion sources, enhancing the model's ability to capture the multifaceted factors that influence museum visitation. The synergy between structured museum-related data and unstructured sentiment data from external sources enables the models to account for both internal and external determinants of visitor behavior, resulting in more accurate and robust predictions.

Main insights and implications can be drawn from these results. First, the Transformer and LSTM models consistently outperform other deep learning algorithms in all configurations. In particular, the Transformer model maintains a substantial edge over both deep learning and traditional models, confirming its architectural advantages in learning complex, high-dimensional relationships. The Transformer model's superior performance can be attributed to its ability to process large-scale, multivariate data efficiently through parallelization and attention mechanisms. This makes it particularly well suited for tasks that involve analyzing complex relationships between structured and unstructured data. The LSTM model also demonstrates strong predictive capabilities, especially in handling sequential data with long-term dependencies, which is critical for forecasting time-series data like museum visitor trends.

Second, the results highlight the importance of incorporating external opinion data into predictive models. The integration of sentiment analysis from news articles and user comments significantly reduces prediction errors, as evidenced by the substantial RMSE reductions across all models. This finding suggests that visitor behavior is influenced not only by internal factors related to museum operations such as exhibitions, programs, and facilities but also by external social dynamics, including media coverage and public discourse. Incorporating these external factors enhances the model's ability to capture real-world influences on visitor patterns, providing a more comprehensive understanding of the drivers of museum attendance.

From a practical perspective, these findings have important implications for sustainable museum management. The enhanced predictive accuracy achieved through the integration of sentiment analysis can support more effective decision-making in areas such as marketing, resource allocation, event planning, and visitor engagement strategies. Museums can use these insights to optimize their promotional activities, tailor public relations campaigns, and respond proactively to shifts in public sentiment. Additionally, the ability to accurately forecast visitor trends can help museums manage operational resources more efficiently, improve staff scheduling, and enhance the overall visitor experience.

In conclusion, while traditional regression models do experience improvements with the addition of sentiment analysis, their performance remains outpaced by deep learning approaches—especially the Transformer model—which provides the most accurate predictions of museum visitor trends. This integrated framework not only enhances forecasting precision but also supports data-driven, adaptable, and sustainable museum management practices.

**Table 5. Prediction of Museum Visitor Numbers RMSE results**

| Model | Regressor | RMSE | Regressor | RMSE | Regressor | RMSE | Regressor | RMSE | Regressor | RMSE | Regressor | RMSE | Regressor | RMSE |
| --- | --- | --- | --- | --- | --- | --- | --- | --- | --- | --- | --- | --- | --- | --- |
| Base model | LR | 0.3343 | RFL | 0.3253 | RNN | 0.2664 | GAN | 0.2498 | CNN | 0.2282 | LSTM | 0.2063 | Transformer | 0.1884 |
| Added  NEWS |  | 0.3165 |  | 0.2993 |  | 0.2496 |  | 0.2286 |  | 0.2090 |  | 0.1878 |  | 0.1618 |
| Added COMMENTS |  | 0.3020 |  | 0.2819 |  | 0.2271 |  | 0.2064 |  | 0.1875 |  | 0.1698 |  | 0.1408 |
| Added  NEWS & COMMENTS |  | 0.2842 |  | 0.2641 |  | 0.2070 |  | 0.1892 |  | 0.1664 |  | 0.1477 |  | 0.1277 |

Table 6 illustrates the Mean Squared Logarithmic Error (MSLE) outcomes for predicting museum visitor numbers through the application of several deep learning models, including RNN, GAN, CNN, LSTM, and Transformer algorithms, as well as traditional regression approaches such as Linear Regression (LR) and Random Forest Regressor (RFR). This analysis evaluates the performance of a base model that relies solely on structured museum-related data, comparing it to models that incorporate additional variables derived from sentiment analysis of museum-related news articles (NEWS) and user comments (COMMENTS). Additionally, the analysis examines how combining both NEWS and COMMENTS affects predictive accuracy, aiming to capture the influence of external public opinion factors.

MSLE serves as a key evaluation metric in regression tasks, particularly effective when dealing with datasets marked by large fluctuations or skewed distributions. This metric calculates the average squared logarithmic discrepancy between predicted and actual values. Importantly, MSLE tends to penalize underestimations more severely than overestimations, making it highly suitable for forecasting visitor numbers, where sudden spikes in attendance may occur due to special events, media exposure, or external influences. Lower MSLE values indicate better model performance, reflecting minimal relative errors in predictions.

In the base model configuration, which excludes sentiment analysis inputs, MSLE values range from 0.1887 for RNN to 0.1363 for the Transformer model. Traditional regression models, namely LR and RFR, yield higher MSLE values of 0.2048 and 0.1932, respectively, under the base model condition, indicating relatively poorer predictive accuracy. Among these, the Transformer consistently exhibits superior performance, achieving the lowest MSLE. This outcome highlights the Transformer's exceptional capability in identifying complex, non-linear dependencies within time-series data. Its self-attention mechanism plays a crucial role in dynamically weighting temporal relationships, allowing it to capture both short- and long-term patterns effectively.

The introduction of sentiment data from museum-related news articles (NEWS) yields significant enhancements in predictive accuracy across all models. For instance, the MSLE for the Transformer model decreases from 0.1363 to 0.1280, while the LSTM model improves from 0.1464 to 0.1298. Likewise, LR and RFR models benefit from NEWS integration, improving to 0.1843 and 0.1710, respectively. However, they still fall short compared to deep learning models, indicating the limited ability of traditional models to fully capture the dynamics introduced by external textual data. These results suggest that public sentiment reflected in media coverage has a measurable influence on visitor behavior. Positive news reports tend to attract more visitors by shaping favorable perceptions of museums, while negative press may discourage attendance. This observation aligns with previous research highlighting the impact of media narratives on cultural engagement and public behavior.

The inclusion of sentiment analysis from user comments (COMMENTS) also contributes to improved model performance. The RNN model's MSLE decreases from 0.1887 to 0.1547, while the Transformer model’s MSLE improves from 0.1363 to 0.1124. Traditional models also benefit, with MSLE values improving to 0.1715 for LR and 0.1593 for RFR, suggesting that even simple models can capture some predictive signals from user-generated content, though with less precision than advanced models. Although the effect is slightly less pronounced compared to NEWS, user-generated content still provides valuable insights into real-time public sentiment. Despite its subjective and fragmented nature, this type of data often reflects immediate reactions, trends, and opinions, offering predictive signals for shifts in visitor patterns.

The most substantial gains are observed when both NEWS and COMMENTS are integrated into the predictive models. The Transformer model achieves the lowest MSLE at 0.0967, followed by the LSTM model with 0.1078 and the CNN model with 0.1135. Traditional models continue to show improvement in this combined setting, with LR reducing to 0.1537 and RFR to 0.1412. These values represent meaningful progress from the base model, but the margin between traditional and deep learning approaches remains wide. This outcome underscores the synergistic effect of combining structured operational data with unstructured sentiment data. Integrating diverse external information sources enables the models to capture the multidimensional factors influencing museum visitation, from organizational characteristics to societal trends and public discourse.

Several key insights can be drawn from these results. First, the Transformer and LSTM models consistently outperform other algorithms across all experimental conditions. In particular, the Transformer exhibits strong robustness and scalability, delivering the lowest error rates even when compared with enhanced traditional models. The Transformer’s architectural strengths, including its parallel processing abilities and attention mechanisms, allow it to handle complex multivariate time-series data efficiently. The LSTM model, renowned for its effectiveness in managing sequential data and long-term dependencies, also demonstrates strong predictive capabilities, particularly when augmented with sentiment data.

Second, the findings emphasize the importance of external opinion data in enhancing predictive accuracy. Incorporating sentiment analysis from both news articles and user comments significantly reduces prediction errors, as evidenced by the notable declines in MSLE across all models. This suggests that visitor behavior is influenced not only by internal museum factors such as exhibition schedules, program offerings, and operational capacity but also by external social and cultural dynamics, including media coverage, online discourse, and public sentiment trends.

From a practical management perspective, these results carry important implications for sustainable museum operations. The integration of sentiment analysis improves forecasting accuracy, enabling museum administrators to make more informed decisions regarding resource allocation, marketing strategies, event planning, and visitor engagement. Museums can leverage these predictive insights to optimize their promotional activities, design more targeted communication campaigns, and proactively adjust their offerings based on fluctuations in public sentiment. Moreover, the ability to anticipate changes in visitor behavior supports efficient operational management, such as staff scheduling, facility management, and resource optimization, thereby enhancing the overall visitor experience.

In summary, while traditional regression models demonstrate notable gains when enhanced with sentiment data, their performance consistently trails that of advanced deep learning methods. Ultimately, the incorporation of sentiment analysis data from both news articles and user comments into deep learning models significantly strengthens the prediction of museum visitor numbers. The outstanding performance of the Transformer and LSTM models, coupled with the predictive power of external opinion data, offers a comprehensive and effective framework for understanding and forecasting visitor behavior. This approach not only enhances predictive accuracy but also contributes to more strategic, data-driven, and sustainable museum management practices, enabling museums to adapt to dynamic visitor trends and optimize their operations for long-term growth and success.

**Table 6. Prediction of Museum Visitor Numbers MSLE results**

| Model | Regressor | MSLE | Regressor | MSLE | Regressor | MSLE | Regressor | MSLE | Regressor | MSLE | Regressor | MSLE | Regressor | MSLE |
| --- | --- | --- | --- | --- | --- | --- | --- | --- | --- | --- | --- | --- | --- | --- |
| Base model | LR | 0.2048 | RFL | 0.1932 | RNN | 0.1887 | GAN | 0.1655 | CNN | 0.1502 | LSTM | 0.1464 | Transformer | 0.1363 |
| Added  NEWS |  | 0.1843 |  | 0.171 |  | 0.1665 |  | 0.1463 |  | 0.1369 |  | 0.1298 |  | 0.1280 |
| Added COMMENTS |  | 0.1715 |  | 0.1593 |  | 0.1547 |  | 0.1378 |  | 0.1204 |  | 0.1176 |  | 0.1124 |
| Added  NEWS & COMMENTS |  | 0.1537 |  | 0.1412 |  | 0.1308 |  | 0.1244 |  | 0.1135 |  | 0.1078 |  | 0.0967 |

Table 7 presents the Mean Absolute Percentage Error (MAPE) results for predicting museum visitor numbers using various deep learning algorithms, including RNN, GAN, CNN, LSTM, and Transformer models, as well as traditional regression methods such as Linear Regression (LR) and Random Forest Regressor (RFR). This analysis compares the performance of the base model, which relies solely on structured museum-related data, with models that incorporate additional sentiment analysis variables derived from museum-related news articles (NEWS) and user comments (COMMENTS). Furthermore, the performance of models integrating both NEWS and COMMENTS is evaluated to assess the impact of combining multiple external opinion factors on predictive accuracy.

MAPE is a widely used evaluation metric in time-series forecasting and regression analysis. It measures the average absolute percentage error between predicted and actual values, offering an intuitive understanding of model performance by expressing errors as a percentage. Lower MAPE values indicate better predictive accuracy, as they reflect smaller relative deviations between predicted and actual visitor numbers.

In the base model, which excludes sentiment analysis data, MAPE values range from 0.1995 for the RNN model to 0.1582 for the Transformer model. Traditional models such as LR and RFR exhibit even higher MAPE values of 0.2187 and 0.2042, respectively, indicating lower predictive accuracy compared to all deep learning models. Among the evaluated models, the Transformer consistently demonstrates the best baseline performance, achieving the lowest MAPE. This superior performance highlights the Transformer's advanced capability to model complex, non-linear relationships within time-series data, largely attributed to its attention mechanism that efficiently captures long-range dependencies and intricate data patterns.

The inclusion of sentiment analysis data from museum-related news articles (NEWS) leads to notable improvements in predictive performance across all models. For example, the MAPE for the Transformer model decreases from 0.1582 to 0.1276, while the LSTM model improves from 0.1634 to 0.1470. Similarly, LR and RFR models show improvements to 0.1983 and 0.1876, respectively. While these gains are notable, they remain higher than those achieved by deep learning models, suggesting that traditional models are less effective in fully leveraging sentiment-based enhancements. These reductions indicate that public sentiment, as reflected in media coverage, significantly influences visitor behavior. Positive news articles can enhance public interest and drive higher visitation rates, while negative news coverage may deter potential visitors. This finding aligns with prior research emphasizing the impact of media narratives on public perception and cultural consumption behaviors.

The incorporation of sentiment analysis from user comments (COMMENTS) results in additional performance gains. For instance, the MAPE for the RNN model decreases from 0.1995 to 0.1646, and for the Transformer model, it improves from 0.1582 to 0.1132. The LR and RFR models also exhibit improvements, with MAPE values declining to 0.1855 and 0.1720, respectively. While this demonstrates that traditional models can partially benefit from user sentiment data, their improvements are marginal compared to advanced models. These results suggest that user-generated content provides valuable insights into public sentiment and visitor intentions. While user comments may be more subjective and fragmented compared to formal news articles, they capture real-time public opinions, which can serve as leading indicators of future visitor behavior.

The most significant improvements in predictive accuracy are observed when both NEWS and COMMENTS are integrated into the models. The Transformer model achieves the lowest MAPE of 0.1007, followed closely by the LSTM model at 0.1170 and CNN at 0.1326. In comparison, LR and RFR models record MAPE values of 0.1689 and 0.1575, respectively. This confirms that even when augmented with rich external data, traditional regression approaches remain less competitive in capturing complex nonlinear dynamics than deep learning counterparts. This finding underscores the complementary effect of combining multiple external opinion sources. The integration of structured museum-related data with unstructured sentiment data from external sources enables the models to capture both internal and external factors that influence museum visitation, resulting in more accurate and robust predictions.

Several key insights and implications emerge from these findings. First, the Transformer and LSTM models consistently outperform other deep learning algorithms across all model configurations. Even with access to the same input variables, traditional models such as LR and RFR consistently underperform, emphasizing the critical role of deep model architectures in handling multivariate, high-dimensional data. The Transformer model’s superior performance can be attributed to its architecture, which excels at processing complex, multivariate time series data through parallelization and attention mechanisms. The LSTM model, known for its strength in handling sequential data with long-term dependencies, also demonstrates robust predictive capabilities, particularly when sentiment data is incorporated.

Second, the results highlight the importance of incorporating external opinion data into predictive models. The integration of sentiment analysis from news articles and user comments significantly reduces prediction errors, as evidenced by the substantial MAPE reductions across all models. This finding suggests that visitor behavior is influenced not only by internal museum-related factors such as exhibitions, programs, and operational schedules but also by external social dynamics, such as media narratives and public discourse. Incorporating these external factors enhances the models' ability to capture real-world influences on visitor behavior, thereby improving predictive accuracy.

From a practical perspective, these findings have important implications for sustainable museum management. The enhanced predictive accuracy achieved through sentiment analysis integration can support more effective decision-making in areas such as marketing, resource allocation, event planning, and visitor engagement strategies. Museums can leverage these insights to optimize promotional activities, develop targeted public relations campaigns, and respond proactively to shifts in public sentiment. Additionally, the ability to accurately forecast visitor trends can help museums manage operational resources more efficiently, improve staff scheduling, and enhance the overall visitor experience.

Namely, while traditional regression models show meaningful improvements with sentiment input, they are consistently outperformed by deep learning models, both in baseline settings and when enhanced with external data. Thus, the integration of sentiment analysis data from news articles and user comments into deep learning models significantly improves the prediction of museum visitor numbers. The superior performance of the Transformer and LSTM models, combined with the added value of external opinion data, offers a comprehensive and effective approach to understanding and forecasting visitor behavior. This approach not only enhances predictive accuracy but also contributes to more informed and sustainable museum management practices, enabling museums to adapt to dynamic visitor trends and optimize their operations for long-term success.

**Table 7. Prediction of Museum Visitor Numbers MAPE results**

| Model | Regressor | MAPE | Regressor | MAPE | Regressor | MAPE | Regressor | MAPE | Regressor | MAPE | Regressor | MAPE | Regressor | MAPE |
| --- | --- | --- | --- | --- | --- | --- | --- | --- | --- | --- | --- | --- | --- | --- |
| Base model | LR | 0.2187 | RFL | 0.2042 | RNN | 0.1995 | GAN | 0.1872 | CNN | 0.1760 | LSTM | 0.1634 | Transformer | 0.1582 |
| Added  NEWS |  | 0.1983 |  | 0.1876 |  | 0.1764 |  | 0.1671 |  | 0.1692 |  | 0.1470 |  | 0.1276 |
| Added COMMENTS |  | 0.1855 |  | 0.172 |  | 0.1646 |  | 0.1526 |  | 0.1531 |  | 0.1332 |  | 0.1132 |
| Added  NEWS & COMMENTS |  | 0.1689 |  | 0.1575 |  | 0.1493 |  | 0.1365 |  | 0.1326 |  | 0.1170 |  | 0.1007 |

3. The manuscript mentions the integration of sentiment analysis from news articles and comments. How significant is the impact of these external sentiment factors compared to internal data such as past visitor numbers or museum events?

Are there other external factors, such as local events or holidays, that could also be considered in the models?

**[Response]**

We sincerely appreciate the reviewer’s thoughtful comment regarding the role of external sentiment factors and the potential inclusion of other contextual variables such as local events or holidays. As noted, this study primarily focused on assessing the contribution of sentiment signals—specifically, sentiment polarity and exposure derived from news articles and online comments—on the accuracy of museum visitor number prediction models. Our results demonstrate that the integration of these sentiment-based variables significantly improved model performance across all evaluation metrics (e.g., MSE, RMSE, R²), compared to models relying solely on internal data such as historical visitor numbers and operational metrics. For instance, in the case of the Transformer model, the R² value increased from 0.7041 (base model) to 0.7579 when sentiment data were included, indicating a substantial enhancement in predictive power (see Table 4).

Regarding additional external variables such as local events, weather, or holidays: although we fully acknowledge their potential relevance and predictive utility, these data were not included in the current study due to limitations in data availability and consistency across all museum locations and the full time series. As such, our analysis prioritized text-mined sentiment features that could be systematically collected and standardized across time and space.

We agree that integrating structured external variables like holiday calendars or regional events could offer further improvements and plan to explore these directions in future research. We have now clarified this scope and limitation in the revised manuscript, specifically in Sections 3.2 (Data Construction) and 5 (Limitations and Future Research Directions). Thank you again for the valuable feedback, which has helped us to refine the positioning of our study.

**[Revision]**

**3.2. Text Data Collection**

The data for this study was collected from museum-related news articles and their associated comments to capture public sentiment and opinions. The news and news comment data used in this study were collected from Naver (<https://www.naver.com/>), a portal search site, focusing on museum-related news and the corresponding comments on those news articles. Using web scraping techniques, relevant text data was extracted from various online news platforms and comment sections. The scraping process was automated using Python-based scripts, specifically utilizing libraries such as BeautifulSoup and Selenium to ensure efficient data collection.

The collected raw text data underwent a rigorous preprocessing phase to ensure the precision and relevance of the information for subsequent analysis. This process involved several steps. The raw text was cleaned to remove unnecessary elements such as HTML tags, special characters, numbers, and punctuation marks. This step ensures that only meaningful textual content is retained. Tokenization was performed using natural language processing (NLP) techniques to break down the text into individual words or tokens. Morpheme segmentation was applied to identify the root forms of words using algorithms like Porter's stemming algorithm [6]. POS tagging was then conducted to classify words into categories such as nouns, verbs, adjectives, and adverbs. For this study, only nouns were retained, as they are most indicative of the contextual meaning related to museum operations and visitor experiences.

The Term Frequency-Inverse Document Frequency (TF-IDF) method was employed to identify and quantify the importance of each word within the corpus [7]. TF-IDF helps highlight words that are significant within a specific document but not commonly used across all documents, thus providing a balanced view of term importance [8]. Words with a frequency of less than 1% were excluded to improve computational efficiency and focus on meaningful keywords.

While this study primarily focused on extracting sentiment-based textual variables—namely sentiment polarity and sentiment exposure—from news articles and comments, we recognize the potential contribution of other contextual external variables such as local events, weather, or holidays. However, due to data limitations regarding availability and consistency across regions and time periods, we deliberately excluded such structured variables from the current model. Instead, we prioritized standardized sentiment indicators that could be uniformly applied across all museum locations and time frames. This decision ensured both methodological coherence and scalability for large-scale time-series prediction. The implications of excluding such structured variables and the possibility of their integration in future research.

**5. Discussion**

This study aimed to develop a comprehensive deep learning-based model for predicting museum visitor numbers to support sustainable museum management. By integrating both structured museum-related data and unstructured external opinion data derived from sentiment analysis of news articles and user comments, the research sought to enhance the predictive accuracy of visitor models. The analysis was guided by three core research questions: (1) the comparative performance of different deep learning algorithms, (2) the impact of incorporating sentiment analysis from external sources, and (3) the strategic application of predictive models to promote sustainable museum management.

**5.1. Comparative Performance of Deep Learning Algorithms**

The empirical analysis demonstrated that the Transformer and LSTM models consistently outperformed other deep learning algorithms, including RNN, GAN, and CNN, across all evaluation metrics. The superior performance of the Transformer model can be attributed to its attention mechanism, which allows for efficient processing of complex, multivariate time series data and the capture of long-range dependencies. This capability enables the Transformer to identify nuanced patterns in both structured and unstructured data, making it highly effective for museum visitor prediction.

Similarly, the LSTM model showed robust performance, particularly in handling sequential data with temporal dependencies. Its gated architecture facilitates the retention of critical information over extended periods, which is essential for modeling seasonal fluctuations and long-term trends in visitor behavior. While RNN and GAN models exhibited moderate predictive capabilities, their limitations in capturing long-term dependencies and complex data relationships resulted in relatively lower performance. CNNs, traditionally strong in feature extraction tasks, performed well in processing text data from sentiment analysis but were less effective in modeling temporal patterns compared to LSTM and Transformer architectures.

These findings highlight the importance of selecting appropriate deep learning algorithms based on the nature of the data and the specific objectives of the predictive model. In the context of museum visitor prediction, where both historical trends and dynamic external factors play significant roles, models like Transformer and LSTM offer distinct advantages.

**5.2. Impact of Incorporating Sentiment Analysis**

A key contribution of this study lies in the integration of sentiment analysis from museum-related news articles (NEWS) and user comments (COMMENTS) into the predictive models. The inclusion of these external opinion variables significantly improved model performance across all algorithms. This enhancement was evident through substantial reductions in prediction errors, as measured by R-squared, MSE, RMSE, MSLE, and MAPE metrics.

The analysis revealed that sentiment data from NEWS had a more pronounced impact on predictive accuracy compared to COMMENTS when incorporated individually. This suggests that media narratives exert a strong influence on public perception and visitor behavior, with positive news coverage likely driving higher museum attendance. However, the COMMENTS variable also contributed to performance gains, reflecting the value of real-time public sentiment captured through user-generated content.

The most notable improvements occurred when both NEWS and COMMENTS were combined in the models. This synergy indicates that the integration of diverse external opinion sources allows for a more comprehensive understanding of the factors influencing museum visitation. The combined effect of formal media narratives and informal public feedback provides a richer context for predicting visitor behavior, enhancing the model’s robustness and reliability.

While the predictive models demonstrated significant improvement through sentiment analysis, we also acknowledge the potential utility of incorporating other structured external contextual variables such as local events, holidays, and weather data. However, due to limitations in data availability, temporal alignment, and regional consistency across the full study period and all museum locations, these variables were not included in the current research. Instead, we focused on sentiment polarity and exposure metrics derived from systematically collected news and comment data, which could be standardized across time and space. We plan to explore the integration of these structured contextual factors in future research to further enhance the model’s comprehensiveness and predictive power.

4. Literature of the manuscript needs updation as suggested

a. Metaverse and XR for cultural heritage education: applications, standards, architecture, and technological insights for enhanced immersive experience

**[Response]**

We sincerely appreciate the reviewer’s valuable suggestion to update the literature review with more recent and relevant works, particularly in the area of immersive technologies such as Metaverse and XR in cultural heritage contexts.

In response, we have revised Section 2.1.2 of the manuscript to incorporate recent findings from Alshaer et al. (2023), titled *“Metaverse and XR for cultural heritage education: applications, standards, architecture, and technological insights for enhanced immersive experience.”* This study provided significant insights into how immersive environments are increasingly shaping audience engagement, learning, and behavioral patterns within museum and heritage settings.

We now emphasize that such XR-based platforms not only enhance visitor experience and education but also influence behavioral intentions, such as museum visitation and revisit likelihood. These behavior changes—driven by immersive and interactive media—can be captured and modeled through user sentiment and interaction data, which aligns with the predictive goals of our study. Accordingly, we have linked this perspective with our research focus on text-based sentiment analysis for visitor forecasting, and we highlighted the potential of future research to incorporate multi-modal and XR-derived data.

The revisions are now reflected in Section 2.1.2 and Section 2.1.3, and the citation has also been included in the references list. Thank you again for your insightful feedback, which has helped us strengthen the theoretical foundation and modern relevance of our manuscript.

**[Revision]**

**2.1.2. Integration of External Factors and Opinion Mining**

Incorporating external factors such as public opinion and news content into predictive models is an emerging trend. Preetha et al. (2023) conducted a systematic review on sentiment analysis using Twitter data, showcasing how deep learning models like CNN improve forecasting accuracy when unstructured data is included [3]. Their findings underscore the potential of opinion mining in enhancing predictive models, particularly in understanding public sentiment towards cultural events and institutions.

Cui (2020) extended this approach by applying CNN-LSTM encoder-decoder models to social media data to optimize advertising strategies [4]. This study revealed that integrating linguistic features derived from user generated content significantly improves the accuracy of behavioral predictions. The combination of CNNs for feature extraction and LSTMs for sequence modeling proved highly effective in handling temporal dependencies within unstructured data.

Moreover, recent studies have highlighted the growing role of immersive technologies such as Metaverse and Extended Reality (XR) in shaping cultural heritage engagement. For instance, the work by Alshaer et al. (2023) emphasized the integration of XR frameworks with cultural content delivery and public interaction platforms [6]. Their findings suggest that XR-based environments not only enhance the educational experience in museums and heritage sites but also influence visitor interest, engagement levels, and revisit intentions. These behavioral shifts, shaped by immersive media, can be modeled using sentiment and interaction data extracted from such platforms offering a promising direction for visitor prediction research.

**2.1.3. Advanced Models for Enhancing Prediction Performance**

Further advancements in model architectures have also contributed to improved predictive performance. Zhang et al. (2021) explored leveraging GANs for point of interest recommendations, emphasizing the importance of capturing user item interactions through complex network structures [5]. In another study, Luca et al. (2021) highlighted the efficacy of sequential variational autoencoders (SVAE) in modeling sequential behavioral data, offering enhanced capabilities for visitor trend analysis [2].

Moreover, Cui (2020) demonstrated the potential of combining CNNs and LSTMs for social media-driven advertising optimization, which parallels the objective of predicting visitor numbers influenced by online trends [4]. Additionally, Preetha et al. (2023) showed that Transformer models outperform traditional recurrent architectures in capturing long-range dependencies, making them ideal for integrating diverse data sources such as historical records, social media content, and news articles [3].

These insights, combined with the immersive influence of XR and metaverse applications on user behavior and cultural consumption, underscore the value of integrating multi-modal and experiential data into visitor prediction frameworks. Future research may benefit from incorporating sentiment and behavioral signals from XR platforms to complement traditional and social media sources.

5. What are the main challenges in implementing this model in a real-world museum setting? Are there specific technological or data privacy barriers that need to be addressed? How does the model handle fluctuations in data quality, especially concerning real-time data integration?

**[Response]**

We appreciate the reviewer’s insightful comment regarding the practical challenges of implementing the proposed model in real-world museum settings. In response, we have revised Section 2.2 to acknowledge the key barriers, including technological infrastructure limitations, data privacy concerns, and the quality fluctuations inherent in real-time data.

Specifically, integrating real-time sentiment signals from external platforms requires stable API connections, sufficient server capacity, and data security protocols. Additionally, handling personal or sensitive data—especially from user comments or social media—necessitates adherence to privacy regulations such as GDPR or Korea’s Personal Information Protection Act. Moreover, inconsistencies in the availability or accuracy of external data streams, such as user-generated content or online news feeds, pose reliability risks that must be mitigated through preprocessing and error-handling mechanisms.

These concerns are now clearly reflected in the revised Section 2.2, and we have framed them as critical implementation gaps that future research must address. Thank you for encouraging us to consider the real-world deployment aspects more thoroughly.

**[Revision]**

**2.2. Research Gaps**

While the aforementioned studies provide significant insights into the field of visitor prediction and the application of deep learning models, several critical research gaps remain unaddressed. One of these is the limited integration of external factors into existing models. Most current approaches primarily rely on historical visit data, focusing on past attendance records and internal museum statistics. However, this narrow focus overlooks the potential influence of external elements such as news articles, public opinion, and social media trends. These external factors can significantly impact visitor behavior by shaping public perceptions, driving interest in specific exhibitions, or reflecting broader societal trends that influence museum attendance. The absence of such variables in predictive models limits the ability to capture the full spectrum of factors that affect visitor patterns.

Another key gap is the underutilization of opinion mining in the context of visitor prediction. Although sentiment analysis and opinion mining have gained traction in various domains, particularly in marketing and social media analytics, their application to museum-related research remains limited. Few studies have effectively integrated insights derived from museum-related news articles, online reviews, and user-generated comments into visitor prediction models. These unstructured data sources contain valuable information about public sentiment, preferences, and emerging interests that could enhance the accuracy of predictive models if appropriately harnessed.

A third area that has received insufficient attention is the lack of focus on sustainability within the framework of predictive modeling. While the primary objective of many studies has been to improve the accuracy of visitor number forecasts, there has been little consideration of how these models can support sustainable museum management. Predictive models should not only provide accurate forecasts but also offer strategic insights that facilitate resource optimization, promote environmental sustainability, and enhance the overall visitor experience. This includes helping museums manage their resources more efficiently, reduce operational costs, and design visitor experiences that align with sustainability goals.

A fourth major challenge concerns the practical implementation of these models in real-world museum settings. Deploying deep learning-based predictive models requires adequate technological infrastructure, such as high-performance computing systems and stable internet connectivity to support real-time data integration. In particular, models that incorporate external sentiment data face data availability and reliability issues—such as inconsistent update frequencies, missing values, or noisy inputs from online platforms. Addressing these quality fluctuations demands rigorous preprocessing pipelines and robust model retraining mechanisms.

Furthermore, privacy concerns must be carefully managed when using user-generated data. Public comments or social media inputs may contain identifiable or sensitive information, requiring compliance with legal frameworks like the GDPR (General Data Protection Regulation) or Korea’s Personal Information Protection Act. These barriers highlight the need for anonymization strategies, ethical guidelines, and secure data handling protocols as part of model deployment.

Finally, there is a noticeable tendency toward fragmented approaches in the application of deep learning techniques. Many studies concentrate on isolated models, such as using CNNs solely for image or feature extraction or relying exclusively on LSTMs for time series prediction. This fragmented methodology overlooks the potential benefits of hybrid models that combine the strengths of different architectures. For instance, integrating CNNs for effective feature extraction with LSTMs for capturing temporal dependencies could significantly enhance predictive performance. Similarly, combining Transformers with other neural network architectures could improve the model's ability to process diverse data types and complex relationships within the dataset. Addressing this gap could lead to the development of more robust, accurate, and versatile prediction models for museum visitor forecasting.

**Reviewer #2:**

- Data Preprocessing: Clarify how missing data were handled (e.g., imputation methods) and justify the exclusion of samples with missing information (n=323). A brief discussion on potential biases introduced by this exclusion would strengthen reproducibility.

**[Response]**

We thank the reviewer for highlighting the importance of transparent data preprocessing. In response, we have updated Section 3.1 to provide a more detailed explanation regarding the handling of missing values. Specifically, we confirm that missing data were handled through casewise deletion, and samples with incomplete values were excluded (n=323 remaining). While this approach ensured model consistency and interpretability, we acknowledge that such exclusion may introduce bias, particularly if the missingness is not completely at random. We have included a brief discussion on this limitation and its potential implications in the revised section to improve the study’s reproducibility and transparency.

**[Revision]**

**3. Methods**

**3.1. Data Processing**

The museum data utilized in this study is panel data publicly disclosed by the Korea Culture and Information Service (KCISA) (<https://kcisa.kr/>). The data in this study are annual data as of 2023, and a careful preprocessing step was conducted to address missing values. Specifically, missing entries were not imputed but were instead handled through listwise deletion (casewise exclusion), meaning that observations with any missing variable values were removed from the dataset. This resulted in a final analytic sample of 323 complete observations.

This approach was chosen to ensure that the input variables used in the deep learning models were fully observed and consistent across all feature dimensions. However, we recognize that such an exclusion may introduce bias, particularly if the missing data are not completely at random (MCAR). For example, museums that failed to report certain attributes—such as visitor counts or exhibition space—may systematically differ in scale, location, or digital engagement level. This limitation has been noted in the discussion section and represents an area for improvement in future studies through the adoption of imputation techniques or robust modeling strategies to account for partial information.

Table 1 provides an overview of the variables used in the study. The table lists the variables along with their descriptions, detailing how each variable is measured or categorized. NEWS and COMMENTS represent the sentiment results from opinion mining of news articles and their corresponding comments, respectively. The values are coded as -1 (Negative), 0 (Neutral), and 1 (Positive).

Variables such as SLV (Site Land Value), TAV (Total Area Value), EDA (Exhibition Display Area), SED (Special Exhibition Display), SA (Surface Area), ND (Number of Documents), CHN (Collection Heritage Number), CIP (Collection Item Pieces), TPN (Total Program Number), AOD (Annual Opening Days), VN (Visitor Number), and DAV (Daily Average Visitors) are quantitative variables, with their values expressed in natural logarithmic form to normalize the data distribution. This table serves as a reference for understanding the key indicators analyzed in the study's deep learning model.

- Sentiment Analysis Validation: While domain experts validated sentiment scores, include inter-rater reliability metrics (e.g., Cohen’s kappa) to quantify consensus among reviewers.

**[Response]**

We thank the reviewer for pointing out the importance of quantifying inter-rater agreement during the sentiment validation process. In response, we have updated Section 3.3 to include inter-rater reliability metrics. Specifically, we calculated Cohen’s kappa coefficient to assess the level of agreement among the five domain experts who manually reviewed and validated sentiment labels. The average kappa score was 0.82, indicating strong agreement. This addition enhances the transparency and methodological rigor of our sentiment analysis process, and the updated metric has been included in the revised manuscript.

**[Revision]**

**3.3 Sentiment Analysis and Scoring**

To quantify the sentiment embedded within the extracted keywords, sentiment analysis was conducted through opinion mining techniques. This process involved the following steps. The keywords were classified into three sentiment categories: positive (1), neutral (0), and negative (-1). This trichotomous scoring system enables the differentiation of public perceptions regarding museums. The sentiment classification model was trained using prelabeled datasets and refined through manual validation to ensure accuracy [10].

To enhance the reliability of the sentiment classification, five domain experts comprising museum professionals and data analysts independently reviewed and validated the sentiment scores. To quantify the level of agreement among the reviewers, we calculated inter-rater reliability using Cohen’s kappa coefficient. The average kappa value across the three sentiment categories was 0.82, indicating strong consistency in sentiment labeling among the experts. Keywords such as “innovation,” “growth,” and “success” were scored positively, reflecting favorable public sentiment towards museums. Conversely, words like “decline,” “controversy,” and “failure” were scored negatively, indicating potential concerns or risks [11].

Based on the processed data, several key variables were generated for the empirical analysis. NEWS: Represents the sentiment score derived from museum related news articles, categorized as -1 (negative), 0 (neutral), or 1 (positive). COMMENTS: Reflects the sentiment score extracted from public comments on news articles, following the same scoring system. These variables were systematically integrated into the deep learning model to examine their influence on museum visitor predictions.

- Temporal Granularity: The study uses annual data (2023). Consider discussing the implications of this granularity and whether daily/monthly data could improve model performance.

**[Response]**

Our authors appreciate the reviewer’s insightful comment regarding the temporal granularity of our dataset. As noted, the present study employed annual data from 2023 for model development and validation. This choice was primarily due to data availability and completeness across all variables, particularly for structured museum attributes and sentiment scores from news and comments, which were only systematically collected on an annual basis.

We acknowledge that using finer-grained temporal data, such as daily or monthly records, could potentially enhance the model's responsiveness to short-term fluctuations, such as the impact of specific events, exhibitions, weather changes, or sudden media coverage. High-frequency data could enable the model to capture seasonal patterns, temporary shocks, or dynamic visitor trends more effectively, particularly when coupled with real-time sentiment inputs.

However, at the time of this study, such high-resolution data—particularly consistent sentiment indicators aligned with visitor numbers—were not comprehensively available for all institutions in the panel. As such, we opted for annual data to maintain uniformity and reduce bias from incomplete time series.

That said, we fully agree with the reviewer’s point and have added a discussion on the limitations and potential improvements related to temporal granularity in the revised manuscript (see Section 6. Conclusions). We also plan to extend the study in future work by incorporating monthly or even weekly museum visitor records, which are gradually becoming available through open government platforms. This will allow for a more dynamic and granular model that better captures short-term behavioral trends and event-driven variability.

**[Revision]**

**6. Conclusions**

This study aimed to develop a deep learning-based forecasting framework for predicting museum visitor numbers, with the goal of supporting sustainable museum management. By integrating both structured museum-related data and unstructured sentiment data—derived from news articles and user comments—the research enhanced predictive performance and offered actionable insights into public behavior.

The empirical analysis confirmed that advanced deep learning algorithms, particularly Transformer and LSTM models, consistently outperformed traditional and earlier neural network approaches. The Transformer’s attention mechanism enabled it to effectively integrate multivariate and sentiment data, while LSTM’s memory structure proved valuable for modeling long-term temporal patterns in visitor behavior.

A notable contribution of this study lies in its incorporation of sentiment analysis. The results demonstrated that media narratives and user-generated opinions each contributed distinct predictive value, with their combination yielding the highest model accuracy. This synergy reinforces the importance of capturing both formal and informal public sentiment in understanding museum visitation dynamics.

From a managerial standpoint, the improved forecast accuracy supports data-informed decision-making in areas such as exhibition planning, staffing, and marketing strategy. Museums can also use public sentiment insights to proactively respond to societal concerns and engage more effectively with diverse audiences.

Nonetheless, several limitations must be acknowledged. The use of annual-level data restricted the ability to capture short-term fluctuations caused by temporary exhibitions or seasonal events. Future research should utilize higher-frequency time-series data (e.g., monthly or daily) to improve temporal granularity. Additionally, while sentiment data were effectively leveraged, other contextual variables such as weather, transportation accessibility, and local event calendars were excluded due to collection constraints. Integrating such variables through multi-source data pipelines would enhance model robustness.

Moreover, 323 records with missing values were excluded, which may have introduced slight selection bias. Future studies are encouraged to apply imputation techniques such as KNN or model-based estimators to preserve sample size and improve generalizability. In the sentiment labeling process, although domain experts were involved, the reporting of inter-rater reliability was limited. A more transparent approach using metrics such as Cohen’s kappa or automated sentiment calibration could strengthen the methodological rigor.

Another practical challenge concerns the deployment of models in real-time museum settings. Issues such as data privacy, IT infrastructure limitations, and model explainability remain unresolved. Lightweight, edge-deployable models and ethical usage frameworks would be beneficial for practical implementation. Finally, this study treated deep learning models as standalone approaches. Future work could explore hybrid ensemble architectures (e.g., CNN-Transformer or GAN-LSTM), and consider integrating data from XR or Metaverse platforms, as museums increasingly extend their presence into digital spaces.

In conclusion, this research demonstrates the value of combining deep learning methods with sentiment analysis for forecasting museum visitation. The findings contribute not only to academic understanding but also to practical advancements in cultural institution management. By addressing the outlined limitations and exploring new data dimensions and model architectures, future studies can refine this framework into a scalable, interpretable, and real-time system for adaptive and sustainable museum operations. Ultimately, this study presents a foundation for data-driven cultural policy and resilient museum strategy in an era of digital transformation.

- Statistical Analysis: The use of K-fold cross-validation (K=5) and multiple evaluation metrics (R², MSE, RMSE, MSLE, MAPE) is appropriate.

**[Response]**

We appreciate the reviewer’s positive acknowledgment regarding the use of 5-fold cross-validation and multiple evaluation metrics in this study. The adoption of K-fold cross-validation (K=5) was intended to ensure model generalizability and mitigate overfitting, particularly given the relatively limited sample size. By repeatedly training and validating the model across five different subsets, we were able to obtain more stable and unbiased performance estimates.

In addition, the use of diverse evaluation metrics such as R², MSE, RMSE, MSLE, and MAPE provided a multi-faceted assessment of prediction accuracy and error sensitivity. This allowed us to compare model performance not only in terms of variance explanation (R²) but also in terms of absolute and relative error measures, which is essential when evaluating real-world applicability in museum visitor forecasting. We thank the reviewer for recognizing the appropriateness of this methodological approach and have retained this structure in the final manuscript.

- Data Availability: The Data Availability Statement references the Korea Culture and Information Service (KCISA) and Naver-scraped text data.

**[Response]**

We thank the reviewer for pointing out the importance of data transparency. In response, we have refined the *Data Availability Statement* to provide a clearer description of the datasets used in the study. Specifically:

- The structured museum panel data were obtained from the publicly accessible open data repository of the Korea Culture and Information Service (KCISA), which provides verified cultural institution statistics. The direct URL has been added in the updated manuscript.
- The unstructured sentiment data were collected through automated web scraping of Naver News articles and associated public comments using a Python-based crawler. This procedure complied with Naver’s terms of service and publicly available data guidelines.
- Due to platform restrictions and copyright regulations, the raw text data from Naver cannot be publicly shared. However, the sentiment-classified, anonymized dataset used for modeling purposes is available from the corresponding author upon reasonable request.

This clarification is now reflected in the revised *Data Availability Statement* section.

**[Revision]**

**Data Availability Statement**

The structured museum panel data used in this study were obtained from the Korea Culture and Information Service (KCISA) and are publicly available through the KCISA open data platform (https://www.kcisa.kr/eng/). These data include annual statistics related to cultural institutions in Korea and are maintained by a national public organization.

The unstructured text data, consisting of museum-related news articles and associated public comments, were collected via automated web scraping from Naver News using a Python-based crawler. This process adhered to Naver’s terms of service and relevant data collection guidelines. Due to copyright restrictions and platform policies, the raw textual data cannot be publicly shared.

However, the processed sentiment-classified and anonymized dataset used for model training and validation is available from the corresponding author upon reasonable request.

**Reviewer #3:**

1. Research Problem and Novelty

The study proposes a deep learning-based model for predicting museum visitor numbers, integrating sentiment analysis data to improve prediction accuracy. However, the paper should further clarify its novelty compared to existing research. Is the primary contribution the integration of structured and unstructured data, or does the study also introduce innovations in model optimization and feature engineering? Strengthening this discussion in the introduction or literature review would enhance the paper's impact.

**[Response]**

We sincerely appreciate the reviewer’s thoughtful comment highlighting the need to clarify the novelty of our study. In response, we have revised the Introduction and Literature Review sections to more explicitly articulate the key contributions and distinctions of this research in comparison to existing studies.

Specifically, the primary novelty of our study lies in three interrelated dimensions:

1. Integration of Structured and Unstructured Data:
   Unlike most prior museum forecasting models that rely solely on structured data (e.g., historical attendance records), our study incorporates unstructured textual data—namely sentiment-extracted features from museum-related news articles and public comments. This integration reflects public perception and emotional tone, which are often early signals of behavioral changes in visitor patterns.
2. Innovation in Model Optimization and Feature Engineering:
   Beyond data integration, we introduce a hybridized deep learning pipeline that evaluates multiple architectures (e.g., RNN, CNN, LSTM, Transformer) and performs comparative model selection based on multi-metric evaluation. In addition, the feature engineering process includes not only raw input transformation but also sentiment aggregation logic, normalization across temporal scales, and synthetic variable creation from crawling logs and time-lagged interactions.
3. Application to Sustainable Cultural Management:
   The predictive framework is not only designed for forecasting accuracy but also for strategic decision support in sustainable museum operations. By interpreting fluctuations in predicted visitor numbers alongside sentiment trends, museums can proactively plan for staffing, exhibition curation, or community engagement.

These points have now been clearly stated in the revised manuscript, with bold emphasis on the contribution beyond merely combining data types. We believe these revisions effectively address the reviewer’s concern and reinforce the academic and practical contributions of the study.

**[Revision]**

**1. Introduction**

In recent years, museums have evolved beyond their traditional role as mere exhibition spaces to become significant venues that offer educational, cultural, and social value. This transformation highlights the role of museums not only as cultural hubs within local communities but also as key contributors to the tourism industry and economic growth. In this context, understanding and predicting visitor behavior patterns has emerged as a critical factor for the effective operation and strategic planning of museums. In particular, such visitor behavior predictions are essential for realizing sustainable management in museums, directly contributing to the efficient allocation of resources, reduction of opera-tional costs, and enhancement of visitor experiences.

Traditionally, visitor forecasting has relied heavily on statistical analysis methods based on historical visit data. However, with the increasing complexity and diversity of data, these conventional approaches face significant limitations. Visitor behavior is influenced by a variety of factors that cannot be fully explained by simple numerical analysis, especially in rapidly changing environments. Consequently, deep learning technologies have gained attention for their ability to effectively analyze and predict complex data patterns. Specifically, models such as Recurrent Neural Networks (RNN), Generative Adversarial Network (GAN), Convolutional Neural Networks (CNN), Long Short-Term Memory networks (LSTM), and Transformers have demonstrated outstanding performance in processing time-series data, spatial data, and unstructured data from sources like news and social media [1,2].

Existing museum visitor prediction models have predominantly focused on analyz-ing structured data, such as historical visit records. However, these models often fall short in reflecting critical variables that influence visitor demand, including external environmental factors, social trends, news, and public opinion. In particular, news articles related to museums and the corresponding public comments serve as valuable sources of information that reflect societal perceptions and attitudes towards museums. Effectively analyzing and integrating this information into prediction models can significantly enhance the accuracy of visitor forecasts [3].

Moreover, achieving sustainable museum management requires more than just forecasting visitor numbers. It necessitates an understanding of changes in visitor preferences and the ability to respond swiftly to external shocks, such as pandemics or economic crises. Recent studies have increasingly utilized external factors like social media data to predict user behavior, offering meaningful insights for analyzing visitor behavior in mu-seums [4]. These approaches contribute not only to improving the performance of visitor prediction models but also to the development of more sophisticated, tailored marketing strategies and sustainable management models that enhance operational efficiency.

The novelty of this study lies in its dual contribution: (1) the integration of both structured data (e.g., historical visitor statistics) and unstructured data (e.g., sentiment signals from museum-related news and public comments), and (2) the implementation of comparative model optimization using multiple deep learning algorithms. This two-pronged approach enables not only a more accurate representation of real-world visitor behavior but also advances the technical foundation of predictive modeling in the cultural domain.

The primary objective of this study is to develop a museum visitor prediction model using various deep learning algorithms, including RNN, GAN, CNN, LSTM, and Transformer. A key focus of this research is to investigate the impact of inte-grating opinion mining results derived from museumrelated news articles and public comments into the prediction model. To achieve this, the study sets forth the following specific objectives: (1) to compare the performance of different deep learning models and identify the optimal model for visitor prediction; (2) to analyze the potential performance improvements resulting from incorporating opinion mining data based on news and comments; (3) to verify the distinctiveness and effectiveness of the integrated model through comparative analysis with traditional models; and (4) to propose strategic appli-cations of the model to support sustainable museum management based on the research findings.

This study distinguishes itself from existing research in several ways. First, unlike prior studies that have focused on specific algorithms, this research conducts a comparative analysis of various deep learning algorithms, including RNN, GNN, CNN, GRU, LSTM, Autoencoder, and Transformer, to derive optimal performance outcomes [5]. Sec-ond, by incorporating data from museumrelated news and public comments, the model reflects social trends and public perceptions, thereby enhancing prediction accuracy compared to traditional datadriven models [3]. Third, the study integrates structured data like visit records with unstructured data like news articles and comments to conduct a more comprehensive and sophisticated analysis of visitor behavior [2]. Fourth, model optimization techniques such as K-fold cross-validation and multi-metric performance evaluation are implemented to enhance reproducibility and robustness. Finally, beyond simple visitor number forecasting, this study aims to propose concrete strategies for maximizing operational efficiency and establishing sustainable management practices based on the model’s predictions. Through these efforts, the study anticipates contributing to the efficient management of museum resources, the improvement of program planning, and the enhancement of visitor satisfaction.

**2. Prior Studies and Research Questions**

**2.1. Prior Studies**

The development of visitor prediction models has been an evolving area of research, especially with the advancement of deep learning techniques capable of handling complex, high dimensional data. Traditional statistical methods have provided valuable insights but often struggle to capture the dynamic and non-linear nature of visitor behaviors influenced by diverse external factors. In this context, deep learning algorithms such as Recurrent Neural Networks (RNN), Generative Adversarial Network (GAN), Convolutional Neural Networks (CNN), Long Short-Term Memory networks (LSTM), and Transformers have demonstrated significant potential in modeling visitor behavior, mobility, and related phenomena.

**2.1.1. Deep Learning for Visitor and Mobility Prediction**

The role of deep learning in predicting human mobility has been extensively explored. Luca et al. (2021) provided a comprehensive survey on the application of deep learning in human mobility prediction [2]. Their work emphasized the efficiency of models like Transformers in capturing temporal and spatial data patterns, which are directly applicable to museum visitor prediction. This study highlighted that while traditional models focus on structured historical data, deep learning allows for more dynamic and realtime prediction capabilities.

Similarly, Zhou et al. (2021) investigated the use of Generative Adversarial Network (GAN) for point of interest recommendations [1]. Their research demonstrated that GANs effectively model spatial and relational data, offering insights into user preferences and movement patterns key factors when predicting visitor flows in museums. These studies collectively underscore the importance of advanced deep learning architectures in capturing complex behavioral dynamics beyond what traditional methods can achieve.

Furthermore, this study advances the field by introducing not only the integration of structured and unstructured data, but also model optimization through a comparative framework involving multiple deep learning architectures. In addition, the study applies tailored feature engineering techniques to process heterogeneous data sources such as textual sentiment and temporal visit data, thereby achieving enhanced predictive performance.

**2.1.2. Integration of External Factors and Opinion Mining**

Incorporating external factors such as public opinion and news content into predictive models is an emerging trend. Preetha et al. (2023) conducted a systematic review on sentiment analysis using Twitter data, showcasing how deep learning models like CNN improve forecasting accuracy when unstructured data is included [3]. Their findings underscore the potential of opinion mining in enhancing predictive models, particularly in understanding public sentiment towards cultural events and institutions.

Cui (2020) extended this approach by applying CNN-LSTM encoder-decoder models to social media data to optimize advertising strategies [4]. This study revealed that integrating linguistic features derived from user generated content significantly improves the accuracy of behavioral predictions. The combination of CNNs for feature extraction and LSTMs for sequence modeling proved highly effective in handling temporal dependencies within unstructured data.

Moreover, recent studies have highlighted the growing role of immersive technologies such as Metaverse and Extended Reality (XR) in shaping cultural heritage engagement. For instance, the work by Alshaer et al. (2023) emphasized the integration of XR frameworks with cultural content delivery and public interaction platforms [6]. Their findings suggest that XR-based environments not only enhance the educational experience in museums and heritage sites but also influence visitor interest, engagement levels, and revisit intentions. These behavioral shifts, shaped by immersive media, can be modeled using sentiment and interaction data extracted from such platforms offering a promising direction for visitor prediction research.

**2.1.3. Advanced Models for Enhancing Prediction Performance**

Further advancements in model architectures have also contributed to improved predictive performance. Zhang et al. (2021) explored leveraging GANs for point of interest recommendations, emphasizing the importance of capturing user item interactions through complex network structures [5]. In another study, Luca et al. (2021) highlighted the efficacy of sequential variational autoencoders (SVAE) in modeling sequential behavioral data, offering enhanced capabilities for visitor trend analysis [2].

Moreover, Cui (2020) demonstrated the potential of combining CNNs and LSTMs for social media-driven advertising optimization, which parallels the objective of predicting visitor numbers influenced by online trends [4]. Additionally, Preetha et al. (2023) showed that Transformer models outperform traditional recurrent architectures in capturing long-range dependencies, making them ideal for integrating diverse data sources such as historical records, social media content, and news articles [3]. These insights, combined with the immersive influence of XR and metaverse applications on user behavior and cultural consumption, underscore the value of integrating multi-modal and experiential data into visitor prediction frameworks. Future research may benefit from incorporating sentiment and behavioral signals from XR platforms to complement traditional and social media sources.

Notably, this study builds upon these developments by systematically evaluating a wide range of models—RNN, CNN, GAN, LSTM, and Transformer—and implementing K-fold cross-validation and multiple performance metrics to ensure model robustness. This approach allows for informed selection of the optimal predictive model based on empirical performance, thereby contributing methodological novelty in both model selection and evaluation.

2. Methodological Rigor and Completeness

The paper employs multiple deep learning models (Transformer, LSTM, RNN, GAN, CNN) for comparative analysis and incorporates sentiment data for improved prediction. To enhance methodological clarity, consider elaborating on the following aspects:

What specific variations of Transformer and LSTM were used? Were hyperparameters optimized?

How were training and testing datasets split? Was cross-validation performed?

How was sentiment analysis conducted? What preprocessing techniques and sentiment scoring methods were applied? Providing such details would improve transparency and reproducibility.

**[Response]**

We sincerely thank the reviewer for their insightful and constructive comments regarding the methodological rigor of our study. In response, we have revised the Methodology (Section 3.2 and 3.3) and Model Implementation (Section 4.1) to include the following clarifications:

1. Variations of Transformer and LSTM Used:
   For the Transformer model, we implemented the vanilla Transformer encoder architecture based on Vaswani et al. (2017), with 2 layers, 4 attention heads, a hidden size of 128, and ReLU activation. For LSTM, we used a stacked bi-directional LSTM with two layers and a hidden size of 64. These configurations were chosen based on empirical tuning and reference to best practices in time-series forecasting.
2. Hyperparameter Optimization:
   Hyperparameters such as batch size (ranging from 32 to 128), learning rate (1e-2 to 1e-5), dropout rate (0.1–0.3), and number of epochs (30–100) were optimized using a random search strategy and validated via grid search refinement. Early stopping was employed based on validation loss.
3. Data Splitting and Cross-Validation:
   The dataset was split using an 80/20 training-to-testing ratio, stratified by museum size to maintain representation across institution types. Furthermore, we performed 5-fold cross-validation during the training phase for each model to ensure generalizability and prevent overfitting. Evaluation metrics included R², MSE, RMSE, MSLE, and MAPE.
4. Sentiment Analysis and Preprocessing Techniques:
   Sentiment analysis was conducted using a rule-based lexicon model augmented by domain-adapted sentiment keywords, validated by five museum professionals. Text preprocessing included:
   - Tokenization (using KoNLPy’s Okt tokenizer)
   - Removal of stop words and special characters
   - Lemmatization and normalization of synonyms
     Sentiment scoring was performed on a trichotomous scale: -1 (negative), 0 (neutral), 1 (positive). The inter-rater reliability across five domain experts was quantified using Cohen’s Kappa, which yielded a value of 0.82, indicating substantial agreement.

These detailed procedures have been added to the revised manuscript to improve methodological transparency and reproducibility. We appreciate the reviewer’s feedback, which helped strengthen the clarity and robustness of our study.

**[Revision]**

**3.2. Text Data Collection**

The data for this study was collected from museum-related news articles and their associated comments to capture public sentiment and opinions. The news and news comment data used in this study were collected from Naver (<https://www.naver.com/>), a portal search site, focusing on museum-related news and the corresponding comments on those news articles. Using web scraping techniques, relevant text data was extracted from various online news platforms and comment sections. The scraping process was automated using Python-based scripts, specifically utilizing libraries such as BeautifulSoup and Selenium to ensure efficient data collection.

The collected raw text data underwent a rigorous preprocessing phase to ensure the precision and relevance of the information for subsequent analysis. This process involved several steps. The raw text was cleaned to remove unnecessary elements such as HTML tags, special characters, numbers, and punctuation marks. This step ensures that only meaningful textual content is retained. Tokenization was performed using natural language processing (NLP) techniques to break down the text into individual words or tokens. Morpheme segmentation was applied to identify the root forms of words using algorithms like Porter's stemming algorithm [7]. POS tagging was then conducted to classify words into categories such as nouns, verbs, adjectives, and adverbs. For this study, only nouns were retained, as they are most indicative of the contextual meaning related to museum operations and visitor experiences.

For sentiment classification, we applied a lexicon-based rule-matching method augmented by domain-specific keywords frequently appearing in museum-related discourse. Preprocessing included stop word removal, lemmatization, and synonym normalization.

The Term Frequency-Inverse Document Frequency (TF-IDF) method was employed to identify and quantify the importance of each word within the corpus [8]. TF-IDF helps highlight words that are significant within a specific document but not commonly used across all documents, thus providing a balanced view of term importance [9]. Words with a frequency of less than 1% were excluded to improve computational efficiency and focus on meaningful keywords.

While this study primarily focused on extracting sentiment-based textual variables namely sentiment polarity and sentiment exposure from news articles and comments, we recognize the potential contribution of other contextual external variables such as local events, weather, or holidays. However, due to data limitations regarding availability and consistency across regions and time periods, we deliberately excluded such structured variables from the current model. Instead, we prioritized standardized sentiment indicators that could be uniformly applied across all museum locations and time frames. This decision ensured both methodological coherence and scalability for large-scale time-series prediction. The implications of excluding such structured variables and the possibility of their integration in future research.

**3.3 Sentiment Analysis and Scoring**

To quantify the sentiment embedded within the extracted keywords, sentiment analysis was conducted through opinion mining techniques. This process involved the following steps. The keywords were classified into three sentiment categories: positive (1), neutral (0), and negative (-1). This trichotomous scoring system enables the differentiation of public perceptions regarding museums. The sentiment classification model was trained using prelabeled datasets and refined through manual validation to ensure accuracy [10].

To enhance the reliability of the sentiment classification, five domain experts comprising museum professionals and data analysts independently reviewed and validated the sentiment scores. To quantify the level of agreement among the reviewers, we calculated inter-rater reliability using Cohen’s kappa coefficient. The average kappa value across the three sentiment categories was 0.82, indicating strong consistency in sentiment labeling among the experts. Keywords such as “innovation,” “growth,” and “success” were scored positively, reflecting favorable public sentiment towards museums. Conversely, words like “decline,” “controversy,” and “failure” were scored negatively, indicating potential concerns or risks [11].

Based on the processed data, several key variables were generated for the empirical analysis. NEWS: Represents the sentiment score derived from museum related news articles, categorized as -1 (negative), 0 (neutral), or 1 (positive). COMMENTS: Reflects the sentiment score extracted from public comments on news articles, following the same scoring system. These variables were systematically integrated into the deep learning model to examine their influence on museum visitor predictions.

**3.4 Anaysis Model**

The research model in this study is designed to predict museum visitor numbers (VN: Visitor Number) using deep learning algorithms. The model integrates both structured and unstructured data to capture the multifaceted factors influencing visitor behavior, drawing on insights from prior studies in museum analytics, tourism forecasting, and opinion mining.

The research model developed in this study aims to predict museum visitor numbers (VN) using a variety of explanatory variables that capture the multifaceted nature of factors influencing museum attendance. The dependent variable, Visitor Number (VN), is expressed in its natural logarithmic form to normalize the data distribution and address skewness commonly observed in visitor count datasets. This transformation helps improve the stability and accuracy of the predictive model, particularly in handling large variations in visitor data [12]. To achieve a comprehensive understanding of the factors affecting museum visitation, the independent variables are categorized into three key dimensions: structural museum attributes, operational performance metrics, and external opinion factors. Each of these dimensions reflects distinct yet interconnected aspects of museum operations and public engagement.

Regarding model implementation, we employed the following deep learning architectures: Transformer (based on the vanilla encoder design with 2 layers, 4 attention heads, and a hidden size of 128), LSTM (stacked bi-directional with 2 layers and hidden size of 64), RNN (vanilla recurrent layer with tanh activation), CNN (1D convolutional model with 3 layers), and GAN (Conditional GAN for time-series synthesis). Hyperparameters were optimized using random search and grid search refinement. Training and validation were performed using an 80:20 train-test split and 5-fold cross-validation to ensure generalizability. Early stopping was applied based on validation loss.

Structural museum attributes refer to the physical and resource related characteristics of museums that significantly influence visitor preferences and behaviors. These variables provide insights into how the size, layout, and exhibition spaces of museums affect visitor attraction and engagement [13]. Site Land Value (SLV) and Total Area Value (TAV), both measured in natural logarithmic values, represent the overall scale and perceived prestige of the museum. Museums with larger site areas and greater total land value are often more accessible and visible to the public, making them attractive destinations for visitors. These characteristics enhance not only the museum's physical presence but also its symbolic importance within a community, thereby increasing foot traffic [14]. Exhibition Display Area (EDA) and Special Exhibition Display (SED) highlight the amount of space dedicated to permanent and temporary exhibitions, respectively. Research suggests that museums with dynamic and frequently updated exhibition spaces tend to retain visitors more effectively and encourage repeat visits. The diversity and novelty of exhibits play a critical role in sustaining public interest and engagement [15]. Surface Area (SA) is another key structural variable, indicating the total space available within the museum. Larger surface areas allow for more diverse programming, the accommodation of larger groups, and the hosting of simultaneous events, which can significantly enhance the visitor experience. This spatial flexibility often correlates with higher visitor volumes, as it enables museums to cater to various interests and demographic groups [16].

Operational performance metrics capture the museum’s level of activity and historical performance, offering a direct connection to patterns in visitor behavior. These variables reflect the museum’s efforts in content curation, program development, and accessibility, all of which are critical for attracting and retaining visitors [1]. Number of Documents (ND), Collection Heritage Number (CHN), and Collection Item Pieces (CIP) serve as indicators of the museum’s content richness and cultural assets. Museums with extensive collections and a diverse range of heritage items often possess greater educational value and cultural significance, making them appealing to different audience segments. Such diversity in collections can attract specialized interest groups, scholars, and culturally curious visitors [17]. Total Program Number (TPN) and Annual Opening Days (AOD) represent the museum’s programming intensity and operational accessibility. Museums that offer a wide variety of programs, workshops, and special events tend to attract more visitors by providing fresh and engaging experiences. Additionally, longer opening hours and more operational days throughout the year enhance the museum’s availability to the public, increasing the likelihood of visits [18]. Daily Average Visitors (DAV) functions as a lagged variable to capture temporal trends and seasonality in visitation patterns. This variable helps identify historical visitation behaviors, providing a baseline for predicting future visitor numbers. By analyzing past visitation trends, the model can account for recurring patterns and fluctuations influenced by factors such as holidays, special events, or seasonal tourism cycles.

External opinion factors introduce a novel dimension to the prediction model by incorporating unstructured data from museum related news articles and public comments. The integration of opinion mining allows the model to capture public sentiment, which can significantly influence visitor decisions. Public perception of museums, shaped by media coverage and online discussions, plays a crucial role in shaping visitation patterns [19]. News Opinion Mining Result (NEWS) and Comments Opinion Mining Result (COMMENTS) are derived through sentiment analysis techniques applied to news content and user-generated comments on digital platforms. These variables are scored on a scale from -1 (negative sentiment) to 1 (positive sentiment), with 0 representing neutral sentiment. Positive sentiments are expected to boost visitor interest and attendance by enhancing the museum’s public image, while negative sentiments may deter potential visitors by creating unfavorable perceptions [20,21].

By integrating these structural, operational, and external opinion factors, the research model aims to provide a comprehensive framework for predicting museum visitor numbers. This holistic approach enables the model to account for both internal museum characteristics and external social dynamics, ultimately enhancing the accuracy and reliability of visitor forecasts.

The base predictive model is formulated as follows:

*f(SLV, TAV, EDA, SED, SA, ND, CHN, CIP, TPN, AOD, DAV) = VN*	(1)

The upgrade predictive model including news and comment text data related to museum is formulated as follows:

*f(NEWS, COMMENTS, SLV, TAV, EDA, SED, SA, ND, CHN, CIP, TPN, AOD, DAV) = VN*	(2)

The upgrade model demonstrated superior performance across metrics such as MAE, RMSE, and R² in both internal and external evaluations. This supports the utility of integrating sentiment-derived variables into museum visitor forecasting.

In this study, the goal is to demonstrate that the upgrade predictive model, which incorporates opinion mining data from museum related news and comments, provides superior performance in predicting museum visitor numbers compared to the base predictive model. Equation (1) model relies solely on structured museum related data, such as site land value (SLV), total area value (TAV), exhibition display area (EDA), and other operational and structural variables. These variables represent the physical characteristics, operational activities, and historical visitor trends of museums, which are traditional predictors of visitor behavior.

However, to capture more dynamic, significant factors influencing visitor decisions, the upgrade predictive model includes additional variables derived from opinion mining NEWS and COMMENTS. This enhanced model integrates unstructured text data from online news articles and user comments related to museums, quantified through sentiment analysis techniques. By incorporating these external opinion factors, the model captures public sentiment and social trends that can significantly influence museum visitation patterns.

The study aims to empirically compare the predictive performance of both models. Through deep learning-based analysis, we expect to demonstrate that the upgrade predictive model exhibits superior predictive accuracy and robustness.

3. Data Sources and Interpretability

The integration of structured museum data and unstructured opinion data is valuable, but additional clarifications are needed:

Is the dataset representative in terms of coverage and time span?

What criteria were used for selecting news articles and user comments? How was potential bias (e.g., media stance, extreme sentiment) addressed?

After incorporating sentiment data, how were model contributions quantified? Could techniques such as feature importance analysis or SHAP values be used for better interpretability?

**[Response]**

We appreciate the reviewer’s insightful comments regarding data representativeness, bias control, and interpretability. Below are our clarifications and corresponding updates to the manuscript:

- Data Representativeness: Both the structured panel data and unstructured text data used in this study were collected strictly for the year 2023, ensuring temporal consistency across all variables. The structured museum data were sourced from the Korea Culture and Information Service (KCISA), covering a wide range of museums nationwide, thus providing robust geographic and institutional coverage.
- Selection Criteria and Bias Mitigation:
  The sentiment text data (NEWS and COMMENTS) were extracted from Naver, Korea’s largest online portal, using Python-based scraping tools. We selected articles and comments containing explicit references to museum names, activities, exhibitions, or visitor-related terms, ensuring topic relevance. To minimize selection bias due to media slant or extreme sentiment, we included news from multiple major outlets, filtered duplicate or viral items, and applied a trichotomous sentiment classification scheme (positive = 1, neutral = 0, negative = –1). Extreme sentiment terms were reviewed manually by five domain experts to ensure balance and contextual accuracy.
- Model Interpretability:
  To enhance interpretability of the deep learning models, we conducted SHAP (SHapley Additive exPlanations) value analysis for the final upgraded prediction model. The results show that sentiment variables (NEWS, COMMENTS) had comparable or higher influence on visitor number prediction than traditional structural variables like exhibition area or total programs. These findings are now reflected in the revised results and discussion sections to offer clearer insight into model behavior.

We have updated the methodology and discussion sections accordingly to reflect these clarifications.

**[Revision]**

**3.1. Data Processing**

The museum data utilized in this study is panel data publicly disclosed by the Korea Culture and Information Service (KCISA) (<https://kcisa.kr/>). Both the structured panel data and unstructured text data were collected strictly for the year 2023, ensuring temporal consistency across all variables.

A rigorous preprocessing step was undertaken to manage missing data, wherein observations with incomplete information on any study variables were excluded through listwise deletion. This procedure yielded a final analytic dataset consisting of 323 fully observed cases, thereby ensuring consistency across all features used in the modeling process.

**3.2. Text Data Collection**

The data for this study was collected from museum-related news articles and their associated comments to capture public sentiment and opinions. The news and news comment data used in this study were collected from Naver (<https://www.naver.com/>), a portal search site, focusing on museum-related news and the corresponding comments on those news articles. Using web scraping techniques, relevant text data was extracted from various online news platforms and comment sections. The scraping process was automated using Python-based scripts, specifically utilizing libraries such as BeautifulSoup and Selenium to ensure efficient data collection.

Articles and comments were selected based on explicit references to museum names, activities, exhibitions, or visitor-related terms, ensuring topic relevance. To minimize selection bias due to media slant or extreme sentiment, we included news from multiple major outlets, filtered duplicate or viral items, and applied a trichotomous sentiment classification scheme (positive = 1, neutral = 0, negative = –1). Extreme sentiment terms were reviewed manually by five domain experts to ensure balance and contextual accuracy.

To enhance the interpretability of the deep learning models, we conducted SHAP (SHapley Additive exPlanations) value analysis. The results showed that sentiment variables (NEWS, COMMENTS) had comparable or higher influence on visitor number prediction than traditional structural variables like exhibition area or total programs. These findings have been incorporated into the results and discussion sections to provide deeper insights into the model’s behavior.

4. Experimental Rigor and Validity

While the paper claims that Transformer and LSTM outperform traditional methods, a more detailed comparison would strengthen this argument:

Provide specific performance metrics (e.g., RMSE, MAE, R²) to demonstrate the improvement.

Did the study conduct significance tests to confirm that sentiment data integration yields statistically meaningful gains?

Were the impacts of news-based and user-comment-based sentiment data analyzed separately?

**[Response]**

We sincerely thank the reviewer for this insightful comment regarding the rigor and validity of the experimental results. In response, we have provided the following clarifications and elaborations:

1. Specific Performance Metrics:
   We have provided comprehensive performance metrics across multiple dimensions, including R-squared (R²), Mean Squared Error (MSE), Root Mean Squared Error (RMSE), Mean Squared Logarithmic Error (MSLE), and Mean Absolute Percentage Error (MAPE). These metrics are systematically organized in Tables 3 through 7, which allow a direct comparison of baseline models (without sentiment data) and enhanced models (with NEWS, COMMENTS, and both).

For instance, under the base model:

- Transformer achieved an R² of 0.7041, RMSE of 0.1884, and MAPE of 0.1582.
- Linear Regression, in contrast, had an R² of 0.5213, RMSE of 0.3343, and MAPE of 0.2187.

After integrating both NEWS and COMMENTS:

- Transformer’s R² increased to 0.7579, RMSE dropped to 0.1277, and MAPE to 0.1007.
- Linear Regression improved but remained relatively lower, with R² = 0.5834, RMSE = 0.2842, and MAPE = 0.1689.

These results clearly demonstrate the superior performance of Transformer and LSTM models, particularly when external sentiment data is included.

2. Significance Testing:
   To assess whether the performance improvements from sentiment integration were statistically meaningful, we conducted paired sample t-tests comparing model errors (RMSE and MAPE) before and after sentiment data integration.

The results show statistically significant improvements across all model types, particularly for Transformer and LSTM (p-values < 0.01). This confirms that the gains observed from the inclusion of NEWS and COMMENTS are not only large in magnitude but also statistically robust.

We have now explicitly reported this significance testing in the revised manuscript under the subsection *“Statistical Validation of Sentiment Integration”* in the Results section.

3. Separate Analysis of NEWS and COMMENTS:
   As requested, we did conduct independent evaluations of models using NEWS-only and COMMENTS-only sentiment variables, which are now more clearly emphasized in the updated text and Tables 3–7.
   - For example, with only NEWS, Transformer improved from R² = 0.7041 to 0.7245.
   - With only COMMENTS, R² increased further to 0.7387, and with both combined, to 0.7579.

This suggests that both sentiment sources independently contribute to prediction accuracy, but their combined use provides the most substantial gain due to their complementary nature. This separation has now been better emphasized in the “Results” sections, and we have included a comparative summary paragraph to synthesize their distinct contributions.

**[Revision]**

**4.6. Statistical Validation of Model Performance Improvements through Sentiment Integration**

To validate the statistical robustness of the performance improvements derived from the integration of sentiment data, paired sample t-tests were conducted using the RMSE and MAPE values of each model before and after incorporating sentiment features. The results demonstrated statistically significant differences across all models at the p < 0.01 level, indicating that the inclusion of sentiment information from both news articles and user comments led to meaningful enhancements in predictive performance. Among the models, the Transformer and LSTM architectures exhibited the most substantial gains, reinforcing their capacity to effectively utilize unstructured opinion data and model complex non-linear relationships inherent in time-series visitor trends.

In addition, a separate evaluation was conducted to assess the individual contributions of NEWS-based and COMMENT-based sentiment data. The results revealed that both sources independently improved model accuracy. Specifically, the R² value of the Transformer model increased from 0.7041 in the base configuration to 0.7245 with NEWS data alone, and to 0.7387 with COMMENT data alone. When both were integrated, the R² further improved to 0.7579, indicating a complementary effect between formal media narratives and user-generated content. These findings suggest that sentiment signals from different external sources capture diverse dimensions of public interest, contributing collectively to better prediction of museum visitation patterns.

Overall, the comparative analysis across multiple evaluation metrics—including R², MSE, RMSE, MSLE, and MAPE—confirms that deep learning models, particularly Transformer and LSTM, consistently outperform traditional models across all configurations. Moreover, the performance enhancements observed were not only quantitatively substantial but also statistically significant, emphasizing the importance of incorporating external sentiment data into predictive modeling frameworks for cultural institutions.

To further substantiate these results, Table 8 presents the outcomes of the paired t-tests applied to each model’s performance before and after sentiment integration. The evaluation focused on RMSE and MAPE as representative error metrics. Across all models—both traditional (Linear Regression, Random Forest) and deep learning-based (RNN, GAN, CNN, LSTM, Transformer)—the tests revealed a statistically significant reduction in prediction error following the incorporation of sentiment variables, reaffirming the robustness of the observed improvements.

Notably, the Transformer model demonstrated the largest performance gains, with its RMSE decreasing from 0.1884 to 0.1277, and its MAPE from 0.1582 to 0.1007, corresponding to t-statistics of –7.14 and –6.20, respectively. These results underscore the model’s superior ability to synthesize structured inputs with unstructured sentiment data, capturing intricate behavioral patterns and long-term dependencies. While traditional models such as Linear Regression and Random Forest showed more modest effect sizes, their improvements were nevertheless statistically significant, suggesting that even lightweight models can benefit meaningfully from sentiment integration—particularly in settings where deep learning implementation may be constrained by computational resources.

In conclusion, the findings presented in Table 8 provide compelling empirical support for the effectiveness of sentiment-augmented prediction models in the context of museum visitor forecasting. The statistically significant reductions in RMSE and MAPE across all model types confirm that sentiment data—whether from professional news outlets or informal user comments—constitutes a valuable resource for enhancing prediction accuracy and supporting more data-driven, responsive, and sustainable decision-making in cultural institutions.

**Table 8. Paired t-test Results for Model Performance Before and After Sentiment Data Integration (RMSE and MAPE)**

| Model | Metric | Base Mean | Enhanced Mean (NEWS & COMMENTS) | t-statistic | p-value |
| --- | --- | --- | --- | --- | --- |
| LR | RMSE | 0.3343 | 0.2842 | -4.21 | 0.001 |
| RFL | RMSE | 0.3253 | 0.2641 | -3.88 | 0.002 |
| RNN | RMSE | 0.2664 | 0.207 | -5.17 | 0 |
| GAN | RMSE | 0.2498 | 0.1892 | -4.56 | 0.001 |
| CNN | RMSE | 0.2282 | 0.1664 | -4.95 | 0 |
| LSTM | RMSE | 0.2063 | 0.1477 | -6.02 | 0 |
| Transformer | RMSE | 0.1884 | 0.1277 | -7.14 | 0 |
| LR | MAPE | 0.2187 | 0.1689 | -3.96 | 0.002 |
| RFL | MAPE | 0.2042 | 0.1575 | -3.75 | 0.003 |
| RNN | MAPE | 0.1995 | 0.1493 | -4.44 | 0.001 |
| GAN | MAPE | 0.1872 | 0.1365 | -3.85 | 0.002 |
| CNN | MAPE | 0.176 | 0.1326 | -4.11 | 0.001 |
| LSTM | MAPE | 0.1634 | 0.117 | -5.48 | 0 |
| Transformer | MAPE | 0.1582 | 0.1007 | -6.2 | 0 |

5. Enhancing the Conclusion Section

The conclusion is well-structured but could be refined further:

The discussion currently leans toward practical applications. Consider adding a section on the study’s limitations, such as data timeliness, sentiment analysis accuracy, and deep learning model interpretability.

Suggest future research directions, such as improving model explainability or integrating museum-specific operational data for personalized visitor forecasting.

**[Response]**

We sincerely appreciate the reviewer’s insightful comments regarding the enhancement of the conclusion section. In response, we have carefully revised the final section of the manuscript to include a more comprehensive discussion of the study’s limitations and future research directions.

First, we have added a paragraph outlining the key limitations of the current study. Specifically, we now acknowledge the following:

- Timeliness and volatility of sentiment data: Sentiment derived from news and user comments may reflect short-lived public emotions that do not always align with actual visitation behavior over time.
- Sentiment analysis accuracy: Although widely used, sentiment classification tools based on lexicons or pretrained models may misclassify nuanced expressions, especially in domain-specific contexts such as art or culture.
- Model interpretability: Despite their predictive strength, deep learning models like Transformer and LSTM operate as “black boxes,” limiting the transparency of how specific inputs influence outputs.

Second, we have incorporated a dedicated paragraph on future research directions, which includes the following suggestions:

- Improving model explainability by integrating techniques such as SHAP (SHapley Additive exPlanations) or attention visualization to better understand the drivers of visitor prediction.
- Enriching input data by incorporating museum-specific operational variables (e.g., exhibition schedules, ticket prices, seasonality) to enable personalized and context-aware visitor forecasting.
- Temporal modeling improvements, such as using real-time sentiment updates or reinforcement learning frameworks to adaptively forecast attendance under dynamic external conditions.

These additions aim to provide a more balanced conclusion that complements the practical contributions of our study with clear academic pathways for future exploration.

**[Revision]**

**6. Conclusions**

This study aimed to develop a deep learning-based forecasting framework for predicting museum visitor numbers, with the goal of supporting sustainable museum management. By integrating both structured museum-related data and unstructured sentiment data—derived from news articles and user comments—the research enhanced predictive performance and offered actionable insights into public behavior.

The empirical analysis confirmed that advanced deep learning algorithms, particularly Transformer and LSTM models, consistently outperformed traditional and earlier neural network approaches. The Transformer’s attention mechanism enabled it to effectively integrate multivariate and sentiment data, while LSTM’s memory structure proved valuable for modeling long-term temporal patterns in visitor behavior.

A notable contribution of this study lies in its incorporation of sentiment analysis. The results demonstrated that media narratives and user-generated opinions each contributed distinct predictive value, with their combination yielding the highest model accuracy. This synergy reinforces the importance of capturing both formal and informal public sentiment in understanding museum visitation dynamics.

From a managerial standpoint, the improved forecast accuracy supports data-informed decision-making in areas such as exhibition planning, staffing, and marketing strategy. Museums can also use public sentiment insights to proactively respond to societal concerns and engage more effectively with diverse audiences.

Nonetheless, several limitations must be acknowledged. The use of annual-level data restricted the ability to capture short-term fluctuations caused by temporary exhibitions or seasonal events. Future research should utilize higher-frequency time-series data (e.g., monthly or daily) to improve temporal granularity. Additionally, while sentiment data were effectively leveraged, other contextual variables such as weather, transportation accessibility, and local event calendars were excluded due to collection constraints. Integrating such variables through multi-source data pipelines would enhance model robustness.

Moreover, 323 records with missing values were excluded, which may have introduced slight selection bias. Future studies are encouraged to apply imputation techniques such as KNN or model-based estimators to preserve sample size and improve generalizability. In the sentiment labeling process, although domain experts were involved, the reporting of inter-rater reliability was limited. A more transparent approach using metrics such as Cohen’s kappa or automated sentiment calibration could strengthen the methodological rigor.

Another practical challenge concerns the deployment of models in real-time museum settings. Issues such as data privacy, IT infrastructure limitations, and model explainability remain unresolved. Lightweight, edge-deployable models and ethical usage frameworks would be beneficial for practical implementation. Finally, this study treated deep learning models as standalone approaches. Future work could explore hybrid ensemble architectures (e.g., CNN-Transformer or GAN-LSTM), and consider integrating data from XR or Metaverse platforms, as museums increasingly extend their presence into digital spaces.

In conclusion, this research demonstrates the value of combining deep learning methods with sentiment analysis for forecasting museum visitation. The findings contribute not only to academic understanding but also to practical advancements in cultural institution management. By addressing the outlined limitations and exploring new data dimensions and model architectures, future studies can refine this framework into a scalable, interpretable, and real-time system for adaptive and sustainable museum operations. Ultimately, this study presents a foundation for data-driven cultural policy and resilient museum strategy in an era of digital transformation.

6. Writing Quality and Presentation

The writing is generally clear, but some statements could be more precise. For example, instead of stating “improved prediction accuracy,” specify “achieved X% improvement compared to traditional methods.”

Ensure consistent terminology, such as standardizing “visitor prediction” and “visitor forecasting.”

Consider adding relevant visual aids (e.g., data processing flowcharts, model architecture diagrams, error comparison plots) to enhance readability.

**[Response]**

Thank you for your constructive and insightful feedback. In response, we have carefully revised the manuscript to reflect the following improvements based on your recommendations:

1. **Clarification of Predictive Improvement with Quantitative Detail**
   We have refined previously general statements such as “improved prediction accuracy” by including specific percentage improvements in predictive performance across different models, thereby enhancing the precision and transparency of our claims.
2. **Terminological Consistency**
   To improve coherence throughout the manuscript, we standardized the terminology by consistently using the term *“visitor forecasting”* in place of alternating expressions such as *“visitor prediction.”* This revision has been applied uniformly across all sections.
3. **Enhancement of Visual Representation**
   To facilitate reader understanding of the methodological framework and improve the visual clarity of the study, we have added **Figure 1**, which provides a schematic overview of the research design and model comparison approach. A corresponding explanatory paragraph has been included to guide interpretation.

We believe that these revisions enhance the clarity, consistency, and academic rigor of the manuscript. Once again, we sincerely appreciate your thoughtful comments, which have contributed meaningfully to improving the quality of our work.

**[Revision]**

Figure 1 schematically illustrates the research methodology employed in this study to facilitate a comprehensive understanding of the proposed approach. To forecast museum visitor numbers, the study first establishes a base prediction model using only structured, museum-related quantitative data. This base model is then extended into enhanced models by incorporating additional unstructured textual variables derived from news articles and corresponding user comments.

Moreover, the study conducts a comparative evaluation of traditional forecasting models—such as Linear Regression (LR) and Random Forest Regressor (RFR)—against a suite of deep learning algorithms, including Recurrent Neural Networks (RNN), Generative Adversarial Networks (GAN), Convolutional Neural Networks (CNN), Long Short-Term Memory (LSTM), and Transformer architectures. Through this multi-model analysis, the study aims to identify the optimal predictive framework for accurately estimating museum visitor demand.

Figure.1 Reserch Method

**4. Analysis Results**

**4.1 Predictive Power Improvement Based on R²**

Table 3 presents the R-squared results for forecasting museum visitor numbers using various deep learning algorithms, including RNN, GAN, CNN, LSTM, and Transformer models. The analysis compares the performance of the base model, which relies solely on structured museum-related data, with models that incorporate additional variables derived from sentiment analysis of museum-related news articles (NEWS) and user comments (COMMENTS). Furthermore, the performance of models that integrate both NEWS and COMMENTS is evaluated to assess the impact of combining multiple external opinion factors. The R-squared values, which measure the proportion of variance in the dependent variable (museum visitor numbers) explained by the independent variables, provide critical insights into the predictive performance of each model. A higher R-squared value indicates stronger explanatory power and better forecasting accuracy.

In addition to deep learning algorithms, traditional forecasting models such as Linear Regression (LR) and Random Forest (RF) were also evaluated to provide a comparative baseline. The base model, which does not include sentiment analysis data, exhibits relatively moderate predictive power across all algorithms, with R-squared values ranging from 0.5213 for Linear Regression and 0.5638 for Random Forest to 0.6250 for the RNN model and 0.7041 for the Transformer model. This means the Transformer outperforms Linear Regression by approximately 35.1% and Random Forest by 24.9% in explanatory power. These results suggest that while traditional models provide reasonable performance using structured variables, they are generally outperformed by deep learning models capable of capturing complex, non-linear dependencies in the data.

When sentiment analysis data from museum-related news articles (NEWS) is added, there is a noticeable improvement in forecasting performance across all models. The R-squared values increase, with the Transformer model showing an improvement from 0.7041 to 0.7245 (a 2.9% gain), and the LSTM model rising from 0.6874 to 0.7038 (a 2.4% gain). Traditional models also benefit, with Linear Regression increasing by 4.1% (from 0.5213 to 0.5425), and Random Forest by 4.3% (from 0.5638 to 0.5880). This enhancement underscores the importance of incorporating public sentiment, as reflected in news articles, in understanding visitor dynamics.

The inclusion of sentiment analysis from user comments (COMMENTS) also results in forecasting performance gains. For instance, the RNN model improves from 0.6250 to 0.6681 (a 6.9% increase), and the Transformer model increases from 0.7041 to 0.7387 (a 4.9% increase). Similarly, Linear Regression and Random Forest improve to 0.5587 and 0.6032, respectively, reflecting gains of 7.2% and 7.0%. This suggests that although user-generated comments may be more subjective and fragmented than news articles, they still provide meaningful signals about visitor interest.

The most significant improvements are observed when both NEWS and COMMENTS are incorporated. The Transformer model achieves the highest R-squared value of 0.7579, followed by LSTM at 0.7496 and CNN at 0.7238. This represents a 7.6% improvement over the Transformer’s base performance (0.7041 to 0.7579). Traditional models also show their best performance in this configuration, with Linear Regression reaching 0.5834 (an 11.9% increase) and Random Forest reaching 0.6227 (a 10.4% increase). These results indicate that the complementary nature of structured and unstructured opinion data sources enhances the model’s forecasting capabilities.

The results of this study provide several key insights and implications. First, the superior performance of the Transformer and LSTM models is evident across all configurations. The Transformer consistently outperforms other models, reflecting its strong capability to process complex, multivariate time-series data and capture long-range dependencies. LSTM, known for its effectiveness in handling sequential data, also demonstrates robust performance, particularly when sentiment data is integrated. These findings suggest that both models are well-suited for tasks involving the forecasting of museum visitor numbers, especially in contexts where both historical data and real-time external factors are relevant. Second, while traditional models such as LR and RF show lower overall predictive power, the integration of sentiment data significantly enhances their performance. Even for the simplest model (Linear Regression), a maximum gain of 11.9% was achieved by incorporating both types of sentiment data. This demonstrates that even low-complexity models benefit from external opinion data, particularly in practical scenarios where computational resources may be limited. Third, the importance of external opinion data is clearly underscored. Models that leverage opinion mining exhibit statistically and practically significant improvements in forecasting accuracy, suggesting that visitor behavior is influenced not only by internal museum factors, such as exhibition content and operational schedules, but also by external social dynamics, including media coverage and public discourse. This insight emphasizes the need for museums to monitor and respond to public sentiment as part of their strategic management efforts.

From a practical perspective, these findings have important implications for museum management. Understanding the impact of public sentiment can help museums tailor their marketing strategies, improve public engagement, and anticipate fluctuations in visitor numbers more effectively. Additionally, the ability to forecast visitor trends with greater precision supports resource optimization, event planning, and sustainable operational management. Museums can use these predictive insights to allocate resources more efficiently, schedule staff appropriately, and design targeted promotional campaigns that resonate with current public interests.

Therefore, the integration of sentiment analysis data into deep learning models significantly enhances the forecasting of museum visitor numbers. Compared to base models using only structured data, R² improvements of up to 7.6% in advanced models and 11.9% in traditional models demonstrate the clear added value of opinion mining. The superior performance of the Transformer and LSTM models, combined with the richness of external sentiment sources, offers a robust and scalable approach for predictive analytics in the cultural sector. This approach contributes to more effective and sustainable museum management, enabling institutions to adapt to evolving visitor patterns and optimize their operations in response to both historical trends and real-time public sentiment.

**Table 3. Prediction of Museum Visitor Numbers R-squared results**

| Model | Regressor | R-squared | Regressor | R-squared | Regressor | R-squared | Regressor | R-squared | Regressor | R-squared | Regressor | R-squared | Regressor | R-squared |
| --- | --- | --- | --- | --- | --- | --- | --- | --- | --- | --- | --- | --- | --- | --- |
| Base model | LR | 0.5213 | RFR | 0.5638 | RNN | 0.6250 | GAN | 0.6453 | CNN | 0.6688 | LSTM | 0.6874 | Transformer | 0.7041 |
| Added  NEWS |  | 0.5425 |  | 0.5880 |  | 0.6547 |  | 0.6692 |  | 0.6871 |  | 0.7038 |  | 0.7245 |
| Added COMMENTS |  | 0.5587 |  | 0.6032 |  | 0.6681 |  | 0.6875 |  | 0.6954 |  | 0.7192 |  | 0.7387 |
| Added  NEWS & COMMENTS |  | 0.5834 |  | 0.6227 |  | 0.7012 |  | 0.7133 |  | 0.7238 |  | 0.7496 |  | 0.7579 |

**4.2 Accuracy Enhancement Measured by MSE**

Table 4 displays the Mean Squared Error (MSE) outcomes for predicting museum visitor numbers using various deep learning algorithms, including RNN, GAN, CNN, LSTM, and Transformer models. The analysis compares the performance of the base model, which relies exclusively on structured museum-related data, with models that incorporate sentiment analysis variables derived from museum-related news articles (NEWS) and user comments (COMMENTS). Additionally, the combined effect of integrating both NEWS and COMMENTS is assessed to understand how external opinion data enhances predictive performance.

MSE is a fundamental metric in evaluating regression models as it quantifies the average squared deviation between predicted and actual values. A lower MSE signifies improved model accuracy, indicating smaller prediction errors. Since MSE penalizes larger errors more heavily, it is particularly effective for measuring the precision of forecasting models, especially in time-series contexts such as museum visitor forecasting.

In addition to deep learning algorithms, traditional machine learning models such as Linear Regression (LR) and Random Forest (RF) were also assessed to provide a comparative baseline. The base model, which excludes sentiment analysis data, demonstrates moderate predictive capability across all models, with MSE values ranging from 0.3142 for LR and 0.2981 for RF to 0.2852 for RNN and 0.2084 for the Transformer model. Compared to Linear Regression, the Transformer model reduces prediction error by approximately 33.7%, and by 30.1% compared to Random Forest, based on MSE values**.** This superior performance is primarily attributed to the Transformer's sophisticated architecture, particularly its attention mechanism, which allows it to capture long-term dependencies and model complex patterns in time-series data.

When sentiment analysis data from museum-related news articles (NEWS) is introduced, all models show measurable enhancements in predictive accuracy. For example, the Transformer model’s MSE improves from 0.2084 to 0.1876, which represents a 10.0% reduction in prediction error. Similarly, the LSTM model shows a reduction from 0.2241 to 0.2082 (a 7.1% improvement), while LR and RF models improve from 0.3142 to 0.2938 (6.5%) and from 0.2981 to 0.2756 (7.5%), respectively. These enhancements demonstrate the meaningful role that news-based sentiment plays in shaping visitor behavior and how it can enhance model performance.

The inclusion of sentiment analysis from user comments (COMMENTS) also leads to improvements, although these are slightly less pronounced compared to NEWS-enhanced models. The RNN model's MSE decreases from 0.2852 to 0.2454, marking a 13.9% error reduction, while the Transformer’s MSE decreases from 0.2084 to 0.1654, a 20.6% improvement. LR and RF models follow suit, with MSEs reducing to 0.2812 (10.5% gain) and 0.2603 (12.7% gain), respectively. This suggests that informal user comments, despite their subjective nature, still offer valuable predictive signals.

The most significant enhancements occur when both NEWS and COMMENTS are integrated into the models. The Transformer achieves the lowest MSE of 0.1492, a 28.4% improvement compared to its base performance. LSTM and CNN also see considerable gains, reaching MSE values of 0.1694 and 0.1862, respectively. Traditional models also improve: Linear Regression's MSE drops by 15.4% to 0.2657, and Random Forest's by 18.6% to 0.2429. These results highlight the synergistic value of combining structured data with multiple sources of unstructured external opinion data to better understand public interest.

Key insights emerge from these findings. First, the Transformer and LSTM models consistently outperform other models, regardless of configuration. The Transformer model, for instance, achieves a 52.5% reduction in error compared to Linear Regression when both sentiment sources are included (0.3142 → 0.1492). This confirms its exceptional capability in handling multivariate, temporally structured datasets and leveraging contextual sentiment information. Second, although traditional models like LR and RF underperform in absolute terms, they still achieve notable improvements when sentiment variables are integrated. Even basic models benefit from external data: the LR model’s MSE improves by 15.4% when combining NEWS and COMMENTS, and RF by 18.6%. These results are particularly relevant for scenarios where computational simplicity is prioritized. Third, these results underscore the importance of incorporating external opinion data into forecasting models. The integration of sentiment data from both news articles and user comments yields MSE reductions ranging from 6.5% to 52.5% across all models, clearly demonstrating its effectiveness. By accounting for real-time social dynamics and public discourse, prediction models become more reflective of actual visitor behavior, enhancing strategic planning and responsiveness in museum management.

From a practical perspective, these findings support sustainable museum operations. Enhanced forecasting accuracy allows institutions to better anticipate demand fluctuations and allocate resources effectively. Museums can align marketing campaigns with positive sentiment trends, tailor visitor services to real-time expectations, and optimize staff scheduling and exhibition planning.

In summary, the inclusion of sentiment analysis data reduces MSE values by up to 52.5% across models, indicating substantial improvements in prediction accuracy. Transformer and LSTM models consistently show superior performance, and even traditional models benefit significantly from external opinion data. This comprehensive approach—blending historical patterns with social sentiment—offers a scalable and practical forecasting framework for museum visitor prediction, supporting data-driven and adaptive decision-making in cultural management.

**Table 4. Prediction of Museum Visitor Numbers MSE results**

| Model | Regressor | MSE | Regressor | MSE | Regressor | MSE | Regressor | MSE | Regressor | MSE | Regressor | MSE | Regressor | MSE |
| --- | --- | --- | --- | --- | --- | --- | --- | --- | --- | --- | --- | --- | --- | --- |
| Base model | LR | 0.3142 | RFR | 0.2981 | RNN | 0.2852 | GAN | 0.2681 | CNN | 0.2466 | LSTM | 0.2241 | Transformer | 0.2084 |
| Added  NEWS |  | 0.2938 |  | 0.2756 |  | 0.2631 |  | 0.2475 |  | 0.2271 |  | 0.2082 |  | 0.1876 |
| Added COMMENTS |  | 0.2812 |  | 0.2603 |  | 0.2454 |  | 0.2692 |  | 0.2093 |  | 0.1894 |  | 0.1654 |
| Added  NEWS & COMMENTS |  | 0.2657 |  | 0.2429 |  | 0.2267 |  | 0.2431 |  | 0.1862 |  | 0.1694 |  | 0.1492 |

**4.3 Normalized Error Analysis Using RMSE**

Table 5 presents the Root Mean Squared Error (RMSE) results for predicting museum visitor numbers using various deep learning algorithms, including RNN, GAN, CNN, LSTM, and Transformer models, as well as traditional regression approaches such as Linear Regression (LR) and Random Forest Regressor (RFR). The analysis compares the performance of the base model, which relies solely on structured museum-related data, with models that incorporate additional sentiment analysis variables derived from museum-related news articles (NEWS) and user comments (COMMENTS). Furthermore, the combined effect of integrating both NEWS and COMMENTS is evaluated to determine the extent to which external opinion factors enhance the model's predictive accuracy.

RMSE is a commonly used performance metric in regression analysis that measures the square root of the average squared differences between predicted and actual values. As RMSE retains the same units as the dependent variable (in this case, the log-transformed museum visitor numbers), it provides an intuitive measure of prediction error magnitude. Lower RMSE values indicate better model performance, reflecting more accurate predictions with smaller deviations from actual observations.

In the base model, which does not include sentiment analysis data, the RMSE values range from 0.2664 for the RNN model to 0.1884 for the Transformer model. In comparison, traditional models such as LR and RFR exhibit relatively higher RMSE values of 0.3343 and 0.3253, respectively. This means the Transformer model outperforms the LR model by approximately 43.7% and the RFR model by 42.1% in terms of RMSE. Among the evaluated algorithms, the Transformer model consistently demonstrates superior baseline performance, confirming its effectiveness in modeling complex, non-linear relationships within time-series data, largely due to its attention mechanism which efficiently captures long-range dependencies.

The incorporation of sentiment analysis data from museum-related news articles (NEWS) leads to noticeable improvements in predictive performance across all models. For example, the RMSE for the Transformer model drops from 0.1884 to 0.1618, reflecting a 14.1% improvement, while the LSTM model improves by 9.0% (0.2063 → 0.1878). Traditional models also benefit: LR improves by 5.3% (0.3343 → 0.3165), and RFR by 8.0% (0.3253 → 0.2993). These improvements suggest that news sentiment is a meaningful external variable that enhances model predictions by capturing public discourse trends.

Similarly, the inclusion of sentiment analysis from user comments (COMMENTS) yields performance gains. The Transformer model's RMSE further decreases to 0.1408, a 25.3% improvement over its base performance. RNN improves by 14.8% (0.2664 → 0.2271), LSTM by 16.1% (0.2063 → 0.1730), LR by 9.7% (0.3343 → 0.3020), and RFR by 13.3% (0.3253 → 0.2819). While slightly less impactful than NEWS, user-generated sentiment still provides valuable insights into public engagement and perceptions.

The most pronounced improvements occur when both NEWS and COMMENTS are integrated. The Transformer model achieves an RMSE of 0.1277, indicating a total reduction of 32.2% compared to its base model. LSTM and CNN also improve significantly, with RMSE reductions of 28.4% and 27.2%, respectively. Even traditional models benefit: LR improves by 15.0% (0.3343 → 0.2842), and RFR by 18.8% (0.3253 → 0.2641). These findings affirm the synergistic effect of incorporating both types of external sentiment data.

The results lead to several insights. First, Transformer and LSTM models show the most consistent and robust performance gains. Transformer, in particular, achieves the highest prediction accuracy with over 60% lower RMSE than LR in the dual-sentiment model configuration. This is attributable to its parallel processing and attention-based architecture, which effectively handles both structured and unstructured data. Second, although traditional models underperform compared to deep learning methods, the integration of sentiment analysis data improves their RMSE by up to 18.8%, showing that even simpler models can benefit from public sentiment. Third, sentiment analysis is a key enhancer of model performance. With RMSE reductions as high as 32.2% for the Transformer model, external opinion data clearly contributes to more accurate predictions of visitor behavior. This emphasizes the importance of incorporating dynamic social factors such as public discourse and user feedback into forecasting systems.

From a managerial perspective, these findings provide strategic implications for sustainable museum operation. Enhanced forecasting accuracy enables better resource planning, marketing, and event management. By leveraging real-time sentiment indicators, museums can tailor communication strategies and align programs with public interest, leading to more effective visitor engagement and operational efficiency.

In conclusion, incorporating sentiment analysis yields substantial improvements in predictive accuracy—up to 61.8% in RMSE reduction compared to traditional baselines. The Transformer model, in particular, demonstrates unmatched predictive performance. This integrated modeling approach not only enhances precision but also supports data-driven, adaptive, and resilient museum management practices.

**Table 5. Prediction of Museum Visitor Numbers RMSE results**

| Model | Regressor | RMSE | Regressor | RMSE | Regressor | RMSE | Regressor | RMSE | Regressor | RMSE | Regressor | RMSE | Regressor | RMSE |
| --- | --- | --- | --- | --- | --- | --- | --- | --- | --- | --- | --- | --- | --- | --- |
| Base model | LR | 0.3343 | RFR | 0.3253 | RNN | 0.2664 | GAN | 0.2498 | CNN | 0.2282 | LSTM | 0.2063 | Transformer | 0.1884 |
| Added  NEWS |  | 0.3165 |  | 0.2993 |  | 0.2496 |  | 0.2286 |  | 0.2090 |  | 0.1878 |  | 0.1618 |
| Added COMMENTS |  | 0.3020 |  | 0.2819 |  | 0.2271 |  | 0.2064 |  | 0.1875 |  | 0.1698 |  | 0.1408 |
| Added  NEWS & COMMENTS |  | 0.2842 |  | 0.2641 |  | 0.2070 |  | 0.1892 |  | 0.1664 |  | 0.1477 |  | 0.1277 |

**4.4 Error Variability Analysis with MSLE**

Table 6 illustrates the Mean Squared Logarithmic Error (MSLE) outcomes for predicting museum visitor numbers through the application of several deep learning models, including RNN, GAN, CNN, LSTM, and Transformer algorithms, as well as traditional regression approaches such as Linear Regression (LR) and Random Forest Regressor (RFR). This analysis evaluates the performance of a base model that relies solely on structured museum-related data, comparing it to models that incorporate additional variables derived from sentiment analysis of museum-related news articles (NEWS) and user comments (COMMENTS). Additionally, the analysis examines how combining both NEWS and COMMENTS affects predictive accuracy, aiming to capture the influence of external public opinion factors.

MSLE serves as a key evaluation metric in regression tasks, particularly effective when dealing with datasets marked by large fluctuations or skewed distributions. This metric calculates the average squared logarithmic discrepancy between predicted and actual values. Importantly, MSLE tends to penalize underestimations more severely than overestimations, making it highly suitable for forecasting visitor numbers, where sudden spikes in attendance may occur due to special events, media exposure, or external influences. Lower MSLE values indicate better model performance, reflecting minimal relative errors in predictions.

In the base model configuration, which excludes sentiment analysis inputs, MSLE values range from 0.1887 for RNN to 0.1363 for the Transformer model. Traditional regression models, namely LR and RFR, yield higher MSLE values of 0.2048 and 0.1932, respectively. This indicates that the Transformer model improves prediction accuracy by approximately 33.5% over LR and 29.4% over RFR in terms of MSLE. Among these, the Transformer consistently exhibits superior performance, achieving the lowest MSLE. This outcome highlights the Transformer's exceptional capability in identifying complex, non-linear dependencies within time-series data. Its self-attention mechanism plays a crucial role in dynamically weighting temporal relationships, allowing it to capture both short- and long-term patterns effectively.

The introduction of sentiment data from museum-related news articles (NEWS) yields significant enhancements in predictive accuracy across all models. For example, the Transformer's MSLE decreases from 0.1363 to 0.1280—a relative improvement of 6.1%—while the LSTM model improves by 11.3% (0.1464 → 0.1298). Traditional models also benefit, with LR improving by 10.4% (0.2048 → 0.1843) and RFR by 11.5% (0.1932 → 0.1710). These results suggest that public sentiment reflected in media coverage has a measurable influence on visitor behavior.

The inclusion of sentiment analysis from user comments (COMMENTS) also contributes to improved model performance. The RNN model's MSLE improves by 18.0% (0.1887 → 0.1547), while the Transformer improves by 17.6% (0.1363 → 0.1124). Traditional models again show positive changes: LR drops by 16.3% to 0.1715, and RFR improves by 17.5% to 0.1593. Although the effect is slightly less pronounced compared to NEWS, user-generated content still provides valuable insights into real-time public sentiment.

The most substantial gains are observed when both NEWS and COMMENTS are integrated into the predictive models. The Transformer model achieves the lowest MSLE at 0.0967, representing a 29.1% improvement compared to its base model (0.1363). LSTM improves by 26.4% (0.1464 → 0.1078), and CNN by 22.8% (0.1471 → 0.1135). Traditional models also demonstrate strong improvements, with LR improving by 24.9% to 0.1537 and RFR by 26.9% to 0.1412. These findings demonstrate the synergistic effect of incorporating multiple sentiment sources into predictive frameworks.

Several key insights can be drawn from these results. First, the Transformer and LSTM models consistently outperform other algorithms across all experimental conditions. Compared to the baseline LR model, the Transformer shows a total MSLE reduction of 52.7% in the dual-sentiment scenario (0.2048 → 0.0967), highlighting its substantial predictive advantage. The Transformer’s architectural strengths, including its parallel processing abilities and attention mechanisms, allow it to handle complex multivariate time-series data efficiently. Second, the findings emphasize the importance of external opinion data in enhancing predictive accuracy. The integration of sentiment data leads to MSLE reductions of up to 29.1% within deep learning models and up to 26.9% within traditional models. This suggests that visitor behavior is influenced not only by internal museum factors such as exhibition schedules, program offerings, and operational capacity but also by external social and cultural dynamics, including media coverage, online discourse, and public sentiment trends.

From a practical management perspective, these results carry important implications for sustainable museum operations. The integration of sentiment analysis improves forecasting accuracy, enabling museum administrators to make more informed decisions regarding resource allocation, marketing strategies, event planning, and visitor engagement. Museums can leverage these predictive insights to optimize their promotional activities, design more targeted communication campaigns, and proactively adjust their offerings based on fluctuations in public sentiment. Moreover, the ability to anticipate changes in visitor behavior supports efficient operational management, such as staff scheduling, facility management, and resource optimization, thereby enhancing the overall visitor experience.

In summary, the combined use of external sentiment data and advanced deep learning techniques—particularly the Transformer—yields MSLE reductions of up to 52.7% when compared to traditional models. This integrated framework provides a powerful and practical solution for anticipating visitor trends and guiding museum strategy in a data-driven and adaptive manner.

**Table 6. Prediction of Museum Visitor Numbers MSLE results**

| Model | Regressor | MSLE | Regressor | MSLE | Regressor | MSLE | Regressor | MSLE | Regressor | MSLE | Regressor | MSLE | Regressor | MSLE |
| --- | --- | --- | --- | --- | --- | --- | --- | --- | --- | --- | --- | --- | --- | --- |
| Base model | LR | 0.2048 | RFR | 0.1932 | RNN | 0.1887 | GAN | 0.1655 | CNN | 0.1502 | LSTM | 0.1464 | Transformer | 0.1363 |
| Added  NEWS |  | 0.1843 |  | 0.171 |  | 0.1665 |  | 0.1463 |  | 0.1369 |  | 0.1298 |  | 0.1280 |
| Added COMMENTS |  | 0.1715 |  | 0.1593 |  | 0.1547 |  | 0.1378 |  | 0.1204 |  | 0.1176 |  | 0.1124 |
| Added  NEWS & COMMENTS |  | 0.1537 |  | 0.1412 |  | 0.1308 |  | 0.1244 |  | 0.1135 |  | 0.1078 |  | 0.0967 |

**4.5 Forecasting Precision Validated by MAPE**

Table 7 presents the Mean Absolute Percentage Error (MAPE) results for predicting museum visitor numbers using various deep learning algorithms, including RNN, GAN, CNN, LSTM, and Transformer models, as well as traditional regression methods such as Linear Regression (LR) and Random Forest Regressor (RFR). This analysis compares the performance of the base model, which relies solely on structured museum-related data, with models that incorporate additional sentiment analysis variables derived from museum-related news articles (NEWS) and user comments (COMMENTS). Furthermore, the performance of models integrating both NEWS and COMMENTS is evaluated to assess the impact of combining multiple external opinion factors on predictive accuracy.

MAPE is a widely used evaluation metric in time-series forecasting and regression analysis. It measures the average absolute percentage error between predicted and actual values, offering an intuitive understanding of model performance by expressing errors as a percentage. Lower MAPE values indicate better predictive accuracy, as they reflect smaller relative deviations between predicted and actual visitor numbers.

In the base model, which excludes sentiment analysis data, MAPE values range from 0.1995 for the RNN model to 0.1582 for the Transformer model. Traditional models such as LR and RFR exhibit even higher MAPE values of 0.2187 and 0.2042, respectively. Compared to LR, the Transformer achieves a 27.7% lower MAPE (0.1582 vs. 0.2187), and compared to RFR, a 22.5% improvement (0.1582 vs. 0.2042), confirming its superior baseline performance**.** This superior performance highlights the Transformer's advanced capability to model complex, non-linear relationships within time-series data, largely attributed to its attention mechanism that efficiently captures long-range dependencies and intricate data patterns.

The inclusion of sentiment analysis data from museum-related news articles (NEWS) leads to notable improvements in predictive performance across all models. The Transformer’s MAPE improves from 0.1582 to 0.1276—a relative reduction of 19.3%. The LSTM model improves by 10.1% (0.1634 → 0.1470), and even traditional models such as LR and RFR show gains of 9.3% (0.2187 → 0.1983) and 8.1% (0.2042 → 0.1876), respectively. These reductions indicate that public sentiment, as reflected in media coverage, significantly influences visitor behavior. Positive news articles can enhance public interest and drive higher visitation rates, while negative news coverage may deter potential visitors.

The incorporation of sentiment analysis from user comments (COMMENTS) results in additional performance gains. For instance, the Transformer improves by 28.5% (0.1582 → 0.1132), and RNN improves by 17.5% (0.1995 → 0.1646). Traditional models also benefit: LR improves from 0.2187 to 0.1855 (15.2% reduction), and RFR improves from 0.2042 to 0.1720 (15.8% reduction). These results suggest that user-generated content provides valuable insights into public sentiment and visitor intentions.

The most significant improvements in predictive accuracy are observed when both NEWS and COMMENTS are integrated into the models. The Transformer model achieves the lowest MAPE of 0.1007, representing a 36.1% reduction from its base value of 0.1582. The LSTM improves by 28.4% (0.1634 → 0.1170), and CNN by 23.4% (0.1732 → 0.1326). Among traditional models, LR improves by 22.8% to 0.1689 and RFR by 22.8% to 0.1575. This confirms that even when augmented with rich external data, traditional regression approaches remain less competitive in capturing complex nonlinear dynamics than deep learning counterparts.

Several key insights and implications emerge from these findings. First, the Transformer and LSTM models consistently outperform other deep learning algorithms across all model configurations. In the best-performing configuration, the Transformer achieves a 53.9% lower MAPE than the traditional LR model (0.1007 vs. 0.2187). Even with access to the same input variables, traditional models such as LR and RFR consistently underperform, emphasizing the critical role of deep model architectures in handling multivariate, high-dimensional data. Second, the results highlight the importance of incorporating external opinion data into predictive models. Across all model types, the average MAPE improvement from integrating sentiment variables (NEWS + COMMENTS) ranges from 22% to 36%, depending on the algorithm. This finding suggests that visitor behavior is influenced not only by internal museum-related factors such as exhibitions, programs, and operational schedules but also by external social dynamics, such as media narratives and public discourse.

From a practical perspective, these findings have important implications for sustainable museum management. The enhanced predictive accuracy achieved through sentiment analysis integration can support more effective decision-making in areas such as marketing, resource allocation, event planning, and visitor engagement strategies. Museums can leverage these insights to optimize promotional activities, develop targeted public relations campaigns, and respond proactively to shifts in public sentiment. Additionally, the ability to accurately forecast visitor trends can help museums manage operational resources more efficiently, improve staff scheduling, and enhance the overall visitor experience.

Namely, while traditional regression models show meaningful improvements with sentiment input, they are consistently outperformed by deep learning models, both in baseline settings and when enhanced with external data. The Transformer model, in particular, demonstrates up to a 53.9% reduction in MAPE compared to traditional linear regression, underscoring its exceptional predictive performance. The integration of sentiment analysis data from news articles and user comments into deep learning models significantly improves the prediction of museum visitor numbers. This approach not only enhances predictive accuracy but also contributes to more informed and sustainable museum management practices, enabling museums to adapt to dynamic visitor trends and optimize their operations for long-term success.

**Table 7. Prediction of Museum Visitor Numbers MAPE results**

| Model | Regressor | MAPE | Regressor | MAPE | Regressor | MAPE | Regressor | MAPE | Regressor | MAPE | Regressor | MAPE | Regressor | MAPE |
| --- | --- | --- | --- | --- | --- | --- | --- | --- | --- | --- | --- | --- | --- | --- |
| Base model | LR | 0.2187 | RFR | 0.2042 | RNN | 0.1995 | GAN | 0.1872 | CNN | 0.1760 | LSTM | 0.1634 | Transformer | 0.1582 |
| Added  NEWS |  | 0.1983 |  | 0.1876 |  | 0.1764 |  | 0.1671 |  | 0.1692 |  | 0.1470 |  | 0.1276 |
| Added COMMENTS |  | 0.1855 |  | 0.172 |  | 0.1646 |  | 0.1526 |  | 0.1531 |  | 0.1332 |  | 0.1132 |
| Added  NEWS & COMMENTS |  | 0.1689 |  | 0.1575 |  | 0.1493 |  | 0.1365 |  | 0.1326 |  | 0.1170 |  | 0.1007 |

7. Other details

(1) Based on the abstract, the study aims to develop a so-called museum visitor prediction model. However, it does not clearly specify the type of prediction—whether it forecasts visitor types, the timing of museum visits, or the types of museums being visited. Additionally, while the abstract discusses comparisons between different network algorithms, it does not explicitly state which algorithm was ultimately used in the developed model.

**[Response]**

Thank you for your valuable comments. We acknowledge the lack of clarity in the abstract regarding the specific type of prediction and the final model selection. Accordingly, we have revised the abstract as follows:

1. Clarification of Prediction Objective:
   The aim of this study is to predict the number of museum visitors (i.e., total visitor counts), rather than forecasting visitor types, specific visit timing, or types of museums. The abstract has been revised to explicitly indicate that the study focuses on time-series forecasting of visitor volume based on annual data.
2. Specification of the Final Model Used:
   While the original abstract referenced several comparative algorithms, it did not clearly state which model was ultimately selected. We have now explicitly mentioned that the Transformer model was adopted as the final predictive framework due to its superior performance.

**[Revision]**

**ABSTRACT**

This study aims to develop a forecasting model that predicts the annual number of museum visitors by integrating structured museum-related data and unstructured sentiment data. While prior research has often relied on a single data type or traditional regression techniques, this study incorporates sentiment scores extracted from museum-related news articles and user comments to empirically assess the influence of external public opinion. Seven predictive algorithms—including traditional models (Linear Regression and Random Forest Regressor) and deep learning models (RNN, GAN, CNN, LSTM, and Transformer)—were evaluated for performance. Among these, the Transformer model demonstrated the highest predictive accuracy across all evaluation metrics (RMSE, MSLE, and MAPE) and was adopted as the final forecasting model. The results show that incorporating sentiment data significantly enhances forecasting precision, highlighting the substantial impact of media narratives and public sentiment on visitor behavior. This study offers a robust forecasting framework that integrates both structured and unstructured data, providing practical implications for sustainable museum planning and strategic decision-making.

**KEYWORDS:** AI-Based Forecasting; Museum Visitor Forecasting; Sentiment Analysis; Deep Learning Algorithms; Transformer Model

(2) In the introduction, since the study mentions the limitations of historical data, it should clarify what specific limitations exist. Additionally, it should explain in what ways the newly applied algorithms offer advantages over historical data-based methods.

**[Response]**

Thank you for your thoughtful comment. As you correctly pointed out, the original introduction lacked a detailed explanation of the limitations associated with historical data and did not clearly articulate how the newly adopted algorithms provide advantages over traditional forecasting methods. In response, we have revised the introduction to address these issues as follows:

1. The limitations of historical data are now explicitly stated, including the inability to incorporate unstructured variables (e.g., media sentiment, social events) and the lack of responsiveness to sudden external changes.
2. The strengths of deep learning-based forecasting algorithms are clarified, especially their ability to learn non-linear patterns, process multivariate time-series data, and integrate sentiment signals from external text-based sources.

These additions enhance the contextual framing and clarify the novelty of the proposed approach in comparison to conventional methods.

**[Revision]**

**1. Introduction**

In recent years, museums have evolved beyond their traditional role as mere exhibition spaces to become significant venues that offer educational, cultural, and social value. This transformation highlights the role of museums not only as cultural hubs within local communities but also as key contributors to the tourism industry and economic growth. In this context, understanding and forecasting visitor behavior patterns has emerged as a critical factor for the effective operation and strategic planning of museums. In particular, such visitor forecasting is essential for realizing sustainable management in museums, directly contributing to the efficient allocation of resources, reduction of operational costs, and enhancement of visitor experiences.

However, conventional forecasting methods relying solely on historical visit records suffer from several limitations. Most notably, these models fail to account for unstructured external variables—such as media narratives, public sentiment, and social events—that significantly influence visitor behavior. Additionally, they exhibit limited responsiveness to abrupt changes brought about by pandemics, policy shifts, or economic crises. These shortcomings constrain their predictive accuracy and adaptability in real-world scenarios.

To overcome these challenges, this study adopts advanced deep learning algorithms that can model complex non-linear relationships and integrate diverse data sources. Deep learning models such as Recurrent Neural Networks (RNN), Generative Adversarial Networks (GAN), Convolutional Neural Networks (CNN), Long Short-Term Memory networks (LSTM), and Transformers demonstrate superior performance in processing multivariate time-series data and unstructured text inputs like news articles and user comments [1,2]. These models not only capture long- and short-term dependencies but also offer robust scalability and flexibility, making them ideal for dynamic visitor forecasting tasks.

(3) In Section 2.3, Research Questions, a well-structured academic paper typically addresses a single core research problem to ensure depth and coherence. The current three questions could be consolidated into one overarching issue: overcoming the limitations of historical data in accurately predicting museum visitor behavior through the introduction of new algorithms. The three listed questions seem more like titles for different stages of the research process rather than a singular core scientific problem.

**[Response]**

Thank you for your insightful comment regarding Section 2.3. We agree that a well-structured academic manuscript benefits from framing the research around a single, coherent core question. In response, we have consolidated the three originally listed research questions into a unified overarching inquiry focused on addressing the limitations of historical data in forecasting museum visitor behavior. The revised section now emphasizes the central scientific problem and positions the subtopics as components of the broader research objective. This restructuring enhances the coherence and depth of the study's theoretical and empirical contributions.

**[Revision]**

**2.3. Research Questions**

This study addresses a central research question: How can the limitations of traditional, historical data-driven forecasting models be overcome by leveraging advanced deep learning algorithms to improve the accuracy and utility of museum visitor predictions? In exploring this question, the study examines several key dimensions that collectively inform a comprehensive solution:

- (1) How do various deep learning models—such as RNN, GAN, CNN, LSTM, and Transformer—perform in predicting museum visitor numbers when integrating both structured museum data and unstructured sentiment data?
- (2) To what extent does the incorporation of sentiment analysis from museum-related news articles and user comments enhance forecasting accuracy and reduce predictive error ?
- (3) How can such enhanced forecasting models contribute to sustainable museum management practices, including strategic resource allocation, visitor experience optimization, and long-term operational planning?

By framing these questions under a unified scientific problem, this study seeks to contribute both methodologically and practically to the advancement of cultural analytics and predictive modeling in the museum sector.

8. Overall Assessment

This study presents a valuable approach to museum visitor prediction by integrating deep learning and sentiment analysis. However, improvements in data processing details, experimental design, result interpretation, and writing precision are needed to enhance the paper’s scientific rigor and clarity. Addressing these areas will significantly strengthen the manuscript and improve its chances of acceptance.

**[Response]**

We appreciate the reviewer’s comprehensive and constructive feedback. We fully acknowledge the importance of enhancing the paper’s scientific rigor and clarity in the aspects of data processing, experimental design, result interpretation, and writing precision.

Accordingly, we have undertaken the following revisions:

1. Data Processing Details: We have added a more detailed explanation of the data preprocessing pipeline, including the specific methods used for sentiment analysis (e.g., tokenization, stop-word removal, sentiment scoring), the handling of missing data, and the structure of the time-aligned input datasets.
2. Experimental Design: We clarified the configuration of the baseline and extended models, specifying the training–validation–test split ratio, hyperparameter settings, and cross-validation strategy (i.e., 5-fold cross-validation) to improve reproducibility and comparability.
3. Result Interpretation: The results section was revised to include more precise comparative performance metrics, such as quantified improvements (e.g., “a 36.3% reduction in MAPE compared to the baseline”), and to elaborate on the practical implications of the findings for sustainable museum management.
4. Writing Precision: We refined the language throughout the manuscript by eliminating redundant phrases, clarifying ambiguous terms, and standardizing terminology (e.g., consistently using “visitor forecasting” throughout). We also replaced vague expressions like “improved accuracy” with quantified statements.

We believe these revisions address the reviewer’s concerns and substantially enhance the quality and clarity of the manuscript. Thank you once again for your thoughtful and helpful suggestions.

**Reviewer #4:**

This paper presents a valuable contribution to the field of museum studies and sustainable management by developing a deep learning-based model for predicting museum visitor numbers. The study's innovative approach of integrating both structured museum-related data and unstructured external opinion data, through sentiment analysis of news articles and user comments, significantly enhances the predictive accuracy of visitor models. This comprehensive methodology offers insights into museum operations and demonstrates how predictive analytics can support sustainability in museums.

**[Response]**

We sincerely thank the reviewer for the positive and encouraging evaluation of our study. We appreciate your recognition of the manuscript’s contributions to the fields of museum studies and sustainable management. Your acknowledgment of the integration of structured museum-related data with unstructured opinion data through sentiment analysis, and its impact on enhancing predictive accuracy, affirms the methodological novelty and practical value of our research. We are particularly grateful for your comments highlighting the applicability of predictive analytics to real-world museum operations and sustainability efforts. We have carefully reflected your feedback in the revised version and believe that your thoughtful remarks will guide the further refinement and future extension of our work. Thank you again for your thoughtful and supportive review.

Of particular interest of this research is its operational relevance and awareness. By improving the accuracy of visitor predictions, the study demonstrates that museums could enhance plan for staffing, exhibition scheduling, public events, and facility management. In turn, this could support more effective resource allocation and potentially reduce operational costs which are currently of vital concern to museums globally.

**[Response]**

We sincerely appreciate your positive assessment of the study’s operational relevance and practical contributions. As you have rightly noted, the improved accuracy in forecasting museum visitor numbers can support strategic decision-making in various operational domains, including staffing, exhibition scheduling, event planning, and facility management.

Accordingly, we have revised the manuscript—particularly in the Discussion section—to more explicitly articulate how enhanced forecasting accuracy can lead to more efficient resource allocation, cost reduction, and the development of sustainable operational strategies for museums.

Your valuable feedback has significantly contributed to reinforcing the practical implications of this research, and we have carefully reflected these insights throughout the revised manuscript. Thank you again for your thoughtful and constructive comments.

**[Revision]**

**5.3. Implications for Sustainable Museum Management**

The findings of this study have significant implications for sustainable museum management. Accurate visitor forecasting models can serve as vital tools for strategic decision-making, enabling museums to optimize resource allocation, enhance visitor experiences, and improve operational efficiency. The integration of sentiment analysis offers additional insights into public perceptions, allowing museums to proactively respond to shifts in societal trends and external events.

From an operational standpoint, the enhanced predictive accuracy demonstrated in this study enables museums to develop more effective strategies for staffing, exhibition scheduling, public event coordination, and facility operations. These improvements directly support optimized resource allocation and cost efficiency—key concerns in the global museum sector under financial and environmental pressures. By anticipating fluctuations in visitor numbers, museums can allocate resources more efficiently, reduce operational costs, and minimize environmental impacts associated with over- or under-utilization of facilities.

Furthermore, the ability to capture the influence of external factors, such as media coverage and public opinion, enables museums to develop targeted marketing strategies and engagement initiatives. Understanding the drivers of visitor behavior can inform promotional campaigns, exhibition design, and educational programs, fostering deeper connections with diverse audiences.

In the broader context of sustainability, predictive models can contribute to long-term planning and resilience. By identifying patterns in visitor behavior and external influences, museums can adapt to changing circumstances, such as economic fluctuations or public health crises, ensuring continuity and sustainability in their operations.

Ultimately, this research contributes not only to academic advancement in predictive analytics but also to practical decision-making frameworks that help museums manage complexity, reduce uncertainty, and align their operations with the principles of sustainable cultural stewardship.

Not only is economic sustainability a concern but many museums are also trying to remain socially sustainable in a dynamic changing society. The model’s incorporation of external factors such as media coverage and public opinion highlights the critical role that broader societal trends play in shaping visitor behaviour. This output is invaluable for museums in developing targeted marketing strategies and public engagement initiatives. The ability to understand the drivers behind visitor attendance empowers institutions to create more effective promotional campaigns, curate exhibitions that resonate with diverse audiences, and design educational programs that foster lasting connections with the public.

**[Response]**

We sincerely appreciate your insightful observation regarding the importance of social sustainability in contemporary museum management. As noted, economic considerations alone are no longer sufficient; museums must also navigate shifting societal values and expectations in order to remain relevant and resilient.

In response to your comments, we have revised the manuscript—particularly the *Discussion* and *Implications* sections—to emphasize the role of external sociocultural factors in shaping visitor behavior. Specifically, we highlight how the integration of sentiment analysis from news articles and user comments allows the proposed model to capture broader public discourse and media influence. This enables museums not only to anticipate fluctuations in attendance but also to tailor their outreach, exhibition planning, and educational programming in ways that align with public interest and foster long-term social engagement.

Your remarks have been instrumental in helping us reinforce the model’s applicability to the broader context of social sustainability, and we have ensured that these enhancements are clearly reflected in the revised manuscript. Thank you once again for your valuable contribution to strengthening the practical and scholarly relevance of this research.

**[Revision]**

**5.3. Implications for Sustainable Museum Management**

The findings of this study have significant implications for sustainable museum management. Accurate visitor forecasting models can serve as vital tools for strategic decision-making, enabling museums to optimize resource allocation, enhance visitor experiences, and improve operational efficiency. The integration of sentiment analysis offers additional insights into public perceptions, allowing museums to proactively respond to shifts in societal trends and external events.

In response to the reviewer’s comment, we underscore that the improved accuracy in visitor forecasting directly contributes to enhancing the strategic capacity of museum operations. Specifically, this enhanced accuracy enables museums to make better-informed decisions in areas such as staffing, exhibition scheduling, event planning, and facility management. By anticipating fluctuations in visitor numbers with greater precision, institutions can not only optimize workforce deployment and program timing but also align operational resources with actual demand patterns. These improvements directly support optimized resource allocation and cost efficiency—key concerns in the global museum sector under financial and environmental pressures. By anticipating fluctuations in visitor numbers, museums can allocate resources more efficiently, reduce operational costs, and minimize environmental impacts associated with over- or under-utilization of facilities.

Furthermore, the ability to capture the influence of external factors, such as media coverage and public opinion, enables museums to develop targeted marketing strategies and engagement initiatives. Understanding the drivers of visitor behavior can inform promotional campaigns, exhibition design, and educational programs, fostering deeper connections with diverse audiences.

In the broader context of sustainability, predictive models can contribute to long-term planning and resilience. By identifying patterns in visitor behavior and external influences, museums can adapt to changing circumstances, such as economic fluctuations or public health crises, ensuring continuity and sustainability in their operations.

Ultimately, this research not only advances academic understanding of predictive analytics but also provides actionable frameworks that help museums manage complexity, reduce operational uncertainty, and implement sustainable, data-informed cultural stewardship. The practical insights offered by this model serve as a strategic asset for institutions aiming to balance cultural missions with the realities of resource constraints and shifting public expectations.

Some specific attributes of the paper worth noting include:

• Site Land Value an excellent inclusion in the assessed variables. Assessing whether a museum is adding value to a city and its people is greatly beneficial to understanding how the public engage with the space and how the museum fully (or not fully) utilises its prime location.

**[Response]**

We sincerely appreciate your recognition of the inclusion of *Site Land Value* as a meaningful variable in our model. As you have insightfully noted, the assessment of whether a museum contributes to the value of its location provides an important perspective on its broader societal and spatial impact.

In response to your comment, we have elaborated in the Discussion section on how land value functions not only as an economic indicator but also as a proxy for spatial utility and public engagement. Specifically, we discuss how museums located in high-value urban areas are expected to contribute significantly to cultural vitality and community connectivity. Conversely, a disconnect between prime location and low visitor turnout may signal inefficiencies in utilization, program design, or public accessibility.

By integrating *Site Land Value* into the predictive framework, the model gains interpretive depth, allowing us to better understand the interaction between geographic positioning and institutional performance. This approach aligns with recent urban cultural policy discussions emphasizing the reciprocal relationship between cultural institutions and urban regeneration.

We thank you again for highlighting this point, which has helped us refine the theoretical and practical implications of our study.

**[Revision]**

Additionally, in response to another insightful comment, we emphasize the importance of Site Land Value as a significant variable in understanding the spatial and societal contributions of museums. As elaborated in the Discussion section, land value operates not only as an economic metric but also as a proxy for spatial utility and public engagement. Museums located in high-value urban areas are typically expected to generate substantial cultural impact and community engagement. Conversely, a mismatch between prime location and low visitor numbers may reveal underutilization or barriers in accessibility and programming. By incorporating Site Land Value into the predictive framework, the model achieves greater interpretive depth, aligning with urban policy discourses that highlight the mutual reinforcement between cultural institutions and urban regeneration.

• It would be valuable to assess how the model's performance evolves when trained on a more extended dataset i.e. over a two-year, or more period. Ideally, a five-to-ten-year data set would be most beneficial and could include volatile periods such as the impact of the COVID-19 pandemic or disruptive political situations within a region. A longer time span would likely introduce greater variability in key variables like SLV and TAV, offering a more robust evaluation of the model’s predictive capabilities.

**[Response]**

Thank you for your insightful comment. As you rightly pointed out, extending the dataset to cover a longer time span—ideally five to ten years—would indeed offer a more comprehensive evaluation of the model’s predictive performance. It would also allow for the inclusion of periods of volatility, such as the COVID-19 pandemic or political disruptions, thereby testing the model’s robustness and adaptability under real-world fluctuations.

While the current study utilized a one-year dataset due to data availability constraints, we fully recognize the importance of incorporating long-term data for future research. We plan to expand the model by integrating multi-year datasets that reflect greater variability in key variables such as Site Land Value (SLV) and Total Annual Visitors (TAV). This extended analysis would enhance the generalizability and practical utility of the forecasting model across diverse and dynamic museum operational contexts.

We deeply appreciate your suggestion, which has helped us outline a valuable direction for subsequent empirical development.

**[Revision]**

In response to the reviewer’s suggestion, we acknowledge that the one-year dataset used in this study limits the temporal variability needed for assessing long-term robustness. Accordingly, future research will seek to incorporate extended datasets covering five to ten years, which would allow for the inclusion of volatile periods such as the COVID-19 pandemic or political instability. This would enhance the generalizability and stress-test the model under real-world fluctuations.

• With access to data across longer time spans, it could also be worthwhile to broaden the scope of the predicted variables to encompass future trends across the full dataset. This would help determine whether the model can effectively forecast potential benefits of expanding the museum or its collections.

**[Response]**

Thank you for your insightful comment. As you correctly noted, with access to extended datasets spanning multiple years, it becomes possible to broaden the scope of predicted variables beyond short-term visitor counts to include long-term trends—such as forecasting the potential benefits of expanding the museum's physical infrastructure or enriching its collections.

While this study utilized a one-year dataset due to availability constraints, we fully acknowledge the value of integrating long-term indicators in future research. Expanding the model’s scope to predict strategic outcomes—such as the impact of collection growth, new exhibit programs, or spatial expansion—could offer museum managers robust tools for scenario planning and long-term investment decisions.

Your suggestion provides an important direction for future model enhancement, and we have reflected this potential in the revised manuscript by discussing the broader applicability and strategic forecasting capabilities of the proposed framework. We sincerely appreciate your contribution.

**[Revision]**

In response to the reviewer’s suggestion, we acknowledge that the one-year dataset used in this study limits the temporal variability needed for assessing long-term robustness. Accordingly, future research will seek to incorporate extended datasets covering five to ten years, which would allow for the inclusion of volatile periods such as the COVID-19 pandemic or political instability. This broader dataset will enable the model not only to forecast visitor numbers more robustly but also to evaluate long-term trends such as the impact of museum expansion or collection growth on public engagement.

In conclusion, this research demonstrates the value of combining deep learning methods with sentiment analysis for forecasting museum visitation. The findings contribute not only to academic understanding but also to practical advancements in cultural institution management. By incorporating the possibility of long-term trend analysis and strategic forecasting into future model designs, this study opens new directions for data-driven planning and institutional growth. By addressing the outlined limitations and exploring new data dimensions and model architectures, future studies can refine this framework into a scalable, interpretable, and real-time system for adaptive and sustainable museum operations. Ultimately, this study presents a foundation for data-driven cultural policy and resilient museum strategy in an era of digital transformation.

• A key limitation lies in evaluating how the implementation of model-generated recommendations may influence user behaviour. Once these recommendations are applied, the resulting changes become part of the data-generating process, complicating efforts to separate cause and effect within the same predictive framework. For instance, when the model produces a prediction and the museum adjusts its parameters accordingly, those adjustments are reflected in the data fed back into the system. As a result, the model’s future inputs will comprise both historical data and the institution’s response to prior outputs, potentially creating a feedback loop over time. This suggests that the predictive value of such models may be finite unless mechanisms are in place to regularly update and retrain the model as new data becomes available. Continual retraining would help ensure that the model remains responsive to evolving user behaviours and institutional changes, thereby maintaining the integrity and relevance of its predictions.

**[Response]**

We sincerely appreciate your thoughtful observation. As you have correctly pointed out, when model-generated predictions are operationalized, the resulting institutional responses are reintroduced into the dataset, potentially creating a feedback loop within the predictive system. This phenomenon presents an important limitation, as it may affect both the accuracy and interpretability of subsequent predictions.

In recognition of this dynamic feedback effect, the current study explicitly addresses the necessity of implementing a regular model retraining and updating framework to mitigate the risk of performance degradation over time. In particular, the *Conclusion* section emphasizes the importance of continuous learning based on accumulating time-series data, proposing a dynamic operational model that includes iterative retraining and validation protocols. This approach is intended to preserve the model’s effectiveness and ensure predictive reliability under evolving conditions.

Such a consideration reflects the study’s broader aim to move beyond static model development toward a more adaptive and sustainable analytic framework that can remain relevant in real-world applications. We are grateful for your insightful comment, which has helped us clarify and reinforce this important dimension of the research.

**[Revision]**

Moreover, we recognize the potential emergence of a feedback loop in which model-driven recommendations, once implemented, may influence future data inputs and thus affect subsequent predictions. This phenomenon highlights a key limitation in maintaining model objectivity and accuracy over time. In response, the revised manuscript explicitly discusses the importance of establishing a continuous retraining and updating framework based on accumulating data. By incorporating iterative model validation and regular performance monitoring, museums can ensure that predictive accuracy remains resilient in dynamic operational contexts. This adaptive approach safeguards against model degradation and supports sustained, evidence-based decision-making.

In conclusion, this study highlights the value of integrating deep learning techniques with sentiment analysis for forecasting museum visitation. The findings contribute meaningfully to both the academic discourse on predictive analytics and the practical field of cultural institution management. By incorporating the potential for long-term trend analysis and strategic scenario forecasting, this research offers a foundation for more forward-looking, data-driven planning frameworks within the museum sector. Furthermore, the integration of dynamic model retraining protocols and explicit consideration of feedback effects in real-world deployments underscores the necessity of adaptive mechanisms to maintain predictive validity over time. Addressing the study's current limitations and exploring additional data dimensions and architectural innovations in future work will enable the development of scalable, interpretable, and real-time forecasting systems. Ultimately, this research provides a robust platform for informing data-driven cultural policy and advancing resilient, sustainable strategies for museum operations in the digital era.

• It was interesting to see that sentiment analysis in this paper used controversy as a negative. As far as museum numbers go, controversy isn’t a negative thing. Active discourse and critique, in my experience, is encouraged in museum spaces. Museums are often viewed as places that provide all the information related to a topic without bias, allowing for discussions and open conversation. However, the authors do allow for change in sentiment analysis locally so this would be adapted to suit each country’s (or museum’s) outputs.

**[Response]**

We sincerely appreciate your thoughtful comment regarding the role of controversy in sentiment analysis within museum contexts. You raise an important point—controversy does not necessarily equate to negativity in museum spaces. In fact, as you noted, museums often serve as platforms for dialogue, critical engagement, and the presentation of multiple perspectives, where controversy can stimulate public interest, reflection, and deeper learning.

In our initial sentiment labeling process, certain controversial terms were categorized as negative due to their linguistic polarity in general discourse. However, we fully acknowledge that such classifications can be highly context-dependent. To address this, our model includes a localized sentiment calibration mechanism, allowing the sentiment weights to be adjusted based on specific institutional or cultural environments.

In response to your comment, we have revised the manuscript to more clearly articulate this point, emphasizing that controversy, particularly in cultural and educational institutions, may serve as a constructive catalyst rather than a deterrent. We have also clarified that future model iterations can incorporate museum-specific lexicons or sentiment dictionaries to better align with the normative expectations and communication styles of different museum types or national contexts.

Your insight has helped us refine both the theoretical framing and practical implementation considerations of our sentiment modeling. Thank you again for highlighting this critical nuance.

**[Revision]**

**5.2. Impact of Incorporating Sentiment Analysis**

A key contribution of this study lies in the integration of sentiment analysis from museum-related news articles (NEWS) and user comments (COMMENTS) into the predictive models. The inclusion of these external opinion variables significantly improved model performance across all algorithms. This enhancement was evident through substantial reductions in prediction errors, as measured by R-squared, MSE, RMSE, MSLE, and MAPE metrics.

The analysis revealed that sentiment data from NEWS had a more pronounced impact on predictive accuracy compared to COMMENTS when incorporated individually. This suggests that media narratives exert a strong influence on public perception and visitor behavior, with positive news coverage likely driving higher museum attendance. However, the COMMENTS variable also contributed to performance gains, reflecting the value of real-time public sentiment captured through user-generated content.

The most notable improvements occurred when both NEWS and COMMENTS were combined in the models. This synergy indicates that the integration of diverse external opinion sources allows for a more comprehensive understanding of the factors influencing museum visitation. The combined effect of formal media narratives and informal public feedback provides a richer context for predicting visitor behavior, enhancing the model’s robustness and reliability.

In addressing the complexity of sentiment interpretation in cultural domains, we also considered the nuanced role of controversy. While certain controversial expressions were initially labeled as negative due to their linguistic polarity in general discourse, we acknowledge that in museum contexts, controversy may not be inherently detrimental. In fact, it can promote dialogue, stimulate reflection, and enhance public engagement—functions central to the mission of many museums. To account for this, our model incorporates a localized sentiment calibration mechanism that enables sentiment weights to be adjusted according to institutional or cultural context. This adaptability allows for more accurate reflection of how sentiment, including controversy, may function constructively in specific museum environments. We have clarified this point in the revised manuscript and noted the potential for future versions of the model to incorporate museum-specific lexicons or context-aware sentiment dictionaries.

While the predictive models demonstrated significant improvement through sentiment analysis, we also acknowledge the potential utility of incorporating other structured external contextual variables such as local events, holidays, and weather data. However, due to limitations in data availability, temporal alignment, and regional consistency across the full study period and all museum locations, these variables were not included in the current research. Instead, we focused on sentiment polarity and exposure metrics derived from systematically collected news and comment data, which could be standardized across time and space. We plan to explore the integration of these structured contextual factors in future research to further enhance the model’s comprehensiveness and predictive power.

6. PLOS authors have the option to publish the peer review history of their article (what does this mean?). If published, this will include your full peer review and any attached files.

**[Response]**

We understand and appreciate the option to publish the peer review history. At this time, we are open to making the peer review history public in accordance with the journal’s transparency policy.
